# High-Entropy Oxide Memristors for Neuromorphic Computing: From Material Engineering to Functional Integration

**DOI:** 10.1007/s40820-025-01891-1

**Published:** 2025-08-25

**Authors:** Jia-Li Yang, Xin-Gui Tang, Xuan Gu, Qi-Jun Sun, Zhen-Hua Tang, Wen-Hua Li, Yan-Ping Jiang

**Affiliations:** 1 School of Physics and Optoelectric Engineering, Guangdong University of Technology, Guangzhou, 510006 People’s Republic of China; 2 Guangdong Provincial Key Laboratory of Sensing Physics and System Integration Applications, Guangdong University of Technology, Guangzhou, 510006 People’s Republic of China

**Keywords:** High-entropy oxides, Memristors, Neuromorphic computing, Configurational entropy, Resistive switching

## Abstract

High-entropy oxides (HEOs) have emerged as a promising class of memristive materials, characterized by entropy-stabilized crystal structures, multivalent cation coordination, and tunable defect landscapes. These intrinsic features enable forming-free resistive switching, multilevel conductance modulation, and synaptic plasticity, making HEOs attractive for neuromorphic computing. This review outlines recent progress in HEO-based memristors across materials engineering, switching mechanisms, and synaptic emulation. Particular attention is given to vacancy migration, phase transitions, and valence-state dynamics—mechanisms that underlie the switching behaviors observed in both amorphous and crystalline systems. Their relevance to neuromorphic functions such as short-term plasticity and spike-timing-dependent learning is also examined. While encouraging results have been achieved at the device level, challenges remain in conductance precision, variability control, and scalable integration. Addressing these demands a concerted effort across materials design, interface optimization, and task-aware modeling. With such integration, HEO memristors offer a compelling pathway toward energy-efficient and adaptable brain-inspired electronics.

## Highlights

- Comprehensive overview of high-entropy oxides (HEOs) in memristive devices, emphasizing their potential in neuromorphic computing and their ability to simulate synaptic plasticity and multilevel conductance modulation.
- Detailed exploration of resistive switching mechanisms in HEO-based memristors, focusing on vacancy migration, phase transitions, and valence-state dynamics, which underpin their performance in brain-inspired electronics.
- Insightful discussion on the challenges and opportunities for integrating HEO-based memristors into large-scale neuromorphic systems, highlighting the need for advancements in material design, interface optimization, and scalability.

## Graphical abstract

**Figure:**
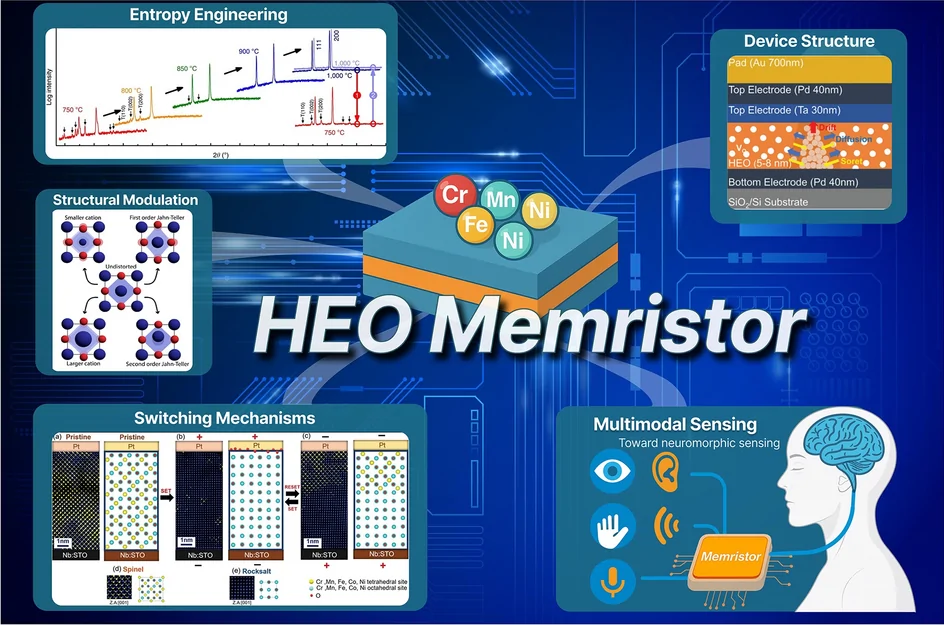

## Introduction

Neuromorphic computing represents a paradigm shift in information processing, aiming to emulate the architecture and dynamic behavior of the human brain [1]. Unlike conventional von Neumann architectures, which are limited by the separation of memory and logic units, neuromorphic systems require devices that can simultaneously store and process information, exhibiting non-volatility, energy efficiency, high integration density, and plasticity-driven adaptability [2–4].

This architectural gap has led to well-known limitations such as the “memory wall” and “power wall” [2, 5, 6]. Memristors, first postulated by Chua [7] and experimentally realized by HP Labs in 2008 through a Pt/TiO_2_/Pt device structure [8], offer a compelling hardware solution by unifying storage and computation within a single, non-volatile component. Beyond their original conception as nonlinear circuit elements, memristors now enable analog conductance modulation, state retention without power, and basic logic functionality (AND, OR, NOT) [9–16]. These capabilities have positioned them as leading candidates for neuromorphic computing, edge intelligence, in-memory architectures, and hardware-level security [4, 17–21].

Memristors, with their simple two-terminal structure and analog conductance modulation, have emerged as promising candidates for artificial synapses [22]. They offer the potential to implement bio-inspired learning rules and support parallel processing in dense memory arrays [17, 18]. Furthermore, their inherent nonlinearity, plasticity, and stochasticity confer robustness, noise tolerance, and adaptability—essential traits for intelligent computing under dynamic environments [23–25].

Despite decades of progress in material development, conventional memristive systems—ranging from binary oxides to perovskites, two-dimensional (2D) materials, and organic compounds—continue to grapple with fundamental performance bottlenecks. Chief among these is limited multilevel stability, non-uniform switching behavior, and inconsistent energy efficiency, all of which hinder the reliable implementation of large-scale neuromorphic architectures [26, 27]. These issues are not merely technical; they reflect deeper limitations in the intrinsic material frameworks upon which such systems are built.

Against this backdrop, HEOs have emerged as a new class of memristive materials. Characterized by entropy-stabilized phase formation, tunable defect chemistries, and multivalent cation coordination, HEOs offer distinct physical advantages for resistive switching and synaptic emulation [28–30]. Their configurational complexity facilitates forming-free operation, high-fidelity multilevel conductance tuning, and enhanced thermal and structural stability—features that directly respond to the bottlenecks of traditional systems [31–33].

While preliminary device-level studies have demonstrated the potential of HEO memristors in supporting both short- and long-term synaptic plasticity, a comprehensive framework linking material design, switching mechanisms, and system-level neuromorphic functionality remains underdeveloped.

**Fig. 1:**
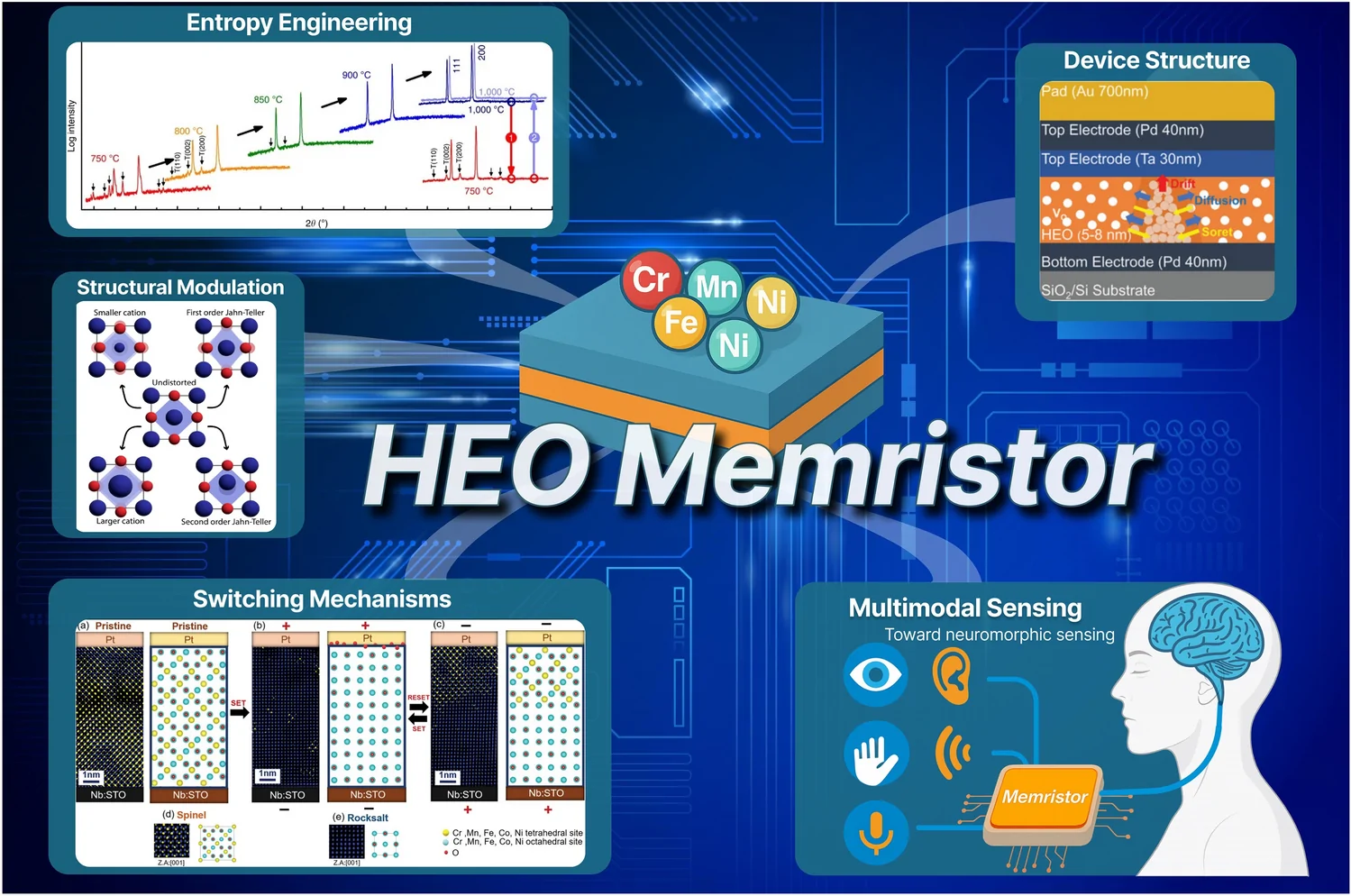
Multiscale overview of HEO memristors, outlining the key themes discussed in this review: Entropy engineering reproduced with permission from Ref. [34], licensed under CC BY 4.0. Structural modulation reproduced with permission from Ref. [29]. Copyright 2023 American Chemical Society. Device Structure reproduced with permission from Ref. [33], licensed under CC BY 4.0. Resistive switching mechanisms reproduced with permission from Ref. [32], Copyright 2023, John Wiley and Sons and Brain-inspired functional integration for neuromorphic sensing

This review systematically addresses this gap by examining HEO-based memristors from the perspectives of materials engineering, device physics, and functional integration. To this end, we construct a multiscale roadmap encompassing four interconnected dimensions: entropy-driven phase stabilization, structural and defect modulation, resistive switching mechanisms, and system-level neuromorphic integration. This conceptual framework is visualized in Fig. 1 and provides the organizational foundation for the sections that follow.

## Materials Foundations of HEOs for Memristive Applications

HEOs represent a unique class of solid-state materials, distinguished by their multicomponent compositions and entropy-stabilized crystal structures. Unlike conventional binary or ternary oxides, HEOs integrate multiple cation species within a single phase, giving rise to local structural disorder, tunable defect chemistries, and enhanced thermodynamic stability. These features are not incidental; they constitute the structural basis for many functional properties critical to memristive behavior, such as resistive switching uniformity, defect migration control, and temperature resilience.

This chapter provides a foundational overview of HEOs from a structural and thermodynamic perspective. We examine how configurational entropy contributes to phase stabilization, how local lattice distortions and chemical heterogeneity influence defect transport, and how these factors can be modulated through synthesis. By establishing these material-level principles, we lay the groundwork for understanding the physical mechanisms by which HEOs enable neuromorphic switching behavior and support device-level functionality in memristive systems.

Before delving into the functional mechanisms of HEOs, it is essential to establish a clear conceptual framework. Section 2.1 defines the terminology, classification criteria, and conceptual evolution of HEOs—laying the groundwork for coherent understanding of their structural behaviors and functional properties.

### Defining and Classifying HEOs

HEOs, a burgeoning subfield within the broader category of high-entropy materials, trace their conceptual origin to the high-entropy alloy (HEA) paradigm. In 2004, Yeh et al. [35] proposed that alloys composed of five or more principal elements in equiatomic or near-equiatomic ratios could form stable, single-phase solid solutions—a notable departure from traditional alloy design strategies that centered around one dominant element with minor dopants. This “multi-principal element + equimolar ratio” principle laid the foundation for the generalized framework of high-entropy materials (HEMs), later extended beyond metallic systems.

A pivotal milestone came in 2015, when Rost et al. [34] demonstrated the entropy stabilization of oxides by synthesizing a rock-salt structured (Co_0.2_Cu_0.2_Mg_0.2_Ni_0.2_Zn_0.2_)O compound. This work marked the first clear evidence that configurational entropy could thermodynamically stabilize a multi-cation oxide into a single-phase structure, thereby introducing the concept of entropy-stabilized oxides (ESOs). Unlike HEAs, HEOs must accommodate ionic and covalent bonding characteristics, often involving mixed-anion lattices to achieve multifunctionality across electronic, magnetic, and optical domains.

Bérardan et al. [36] subsequently coined the term “HEOs” and explored their transport and dielectric properties in greater detail. They found that through compositional tuning, the electrical conductivity of HEOs could vary across a wide range (10^–8^ to 10^–3^ S cm^−1^), while their dielectric constants could reach as high as 10^4^–10^5^ orders of magnitude beyond traditional oxides such as Al_2_O_3_ (*ε*_r_ ≈ 9). These findings highlighted the promise of HEOs for next-generation functional electronics, including neuromorphic computing.

**Fig. 2:**
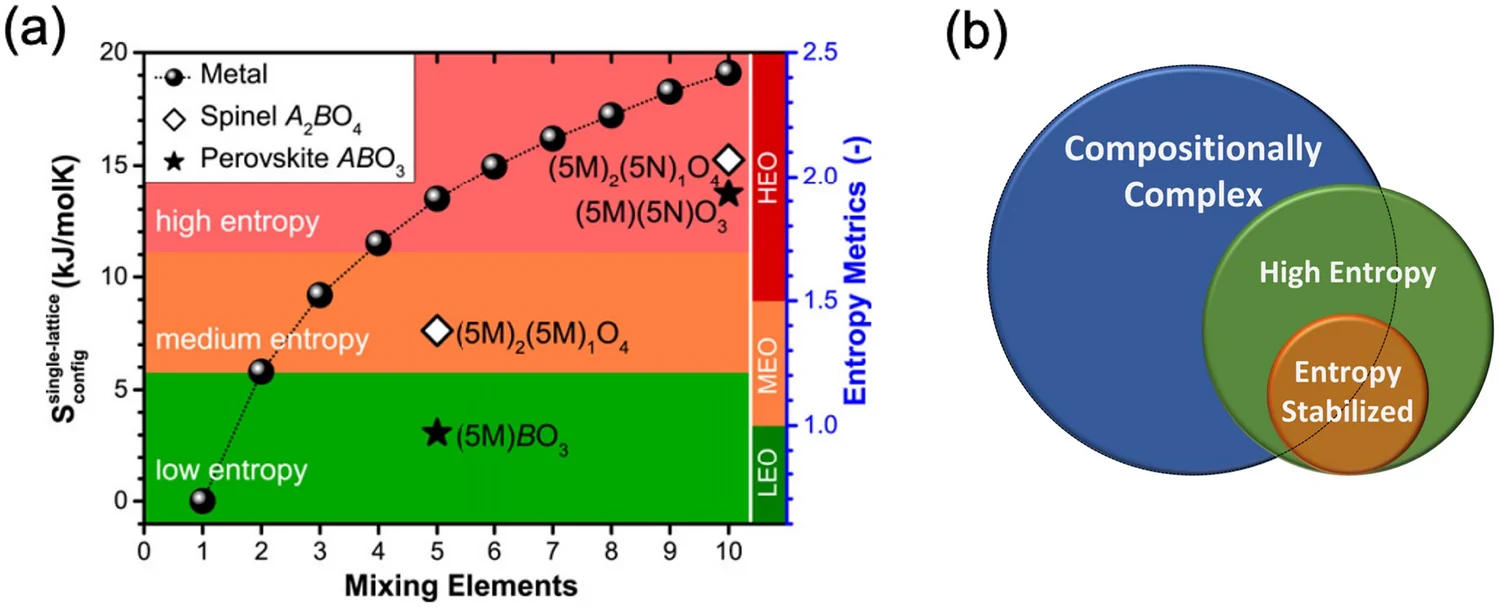
Defining and classifying HEOs. **a** Plot of *S*_config_ versus the number of mixing elements, classifying materials into high, medium, and low entropy categories.** b** Venn diagram showing the relationship between CCMs, HEOs, and ESOs. Reproduced with permission from Ref. [38], licensed under CC BY 4.0

Despite this progress, early definitions of HEOs were often vague sometimes equating the presence of five or more elements with high entropy, without quantitatively evaluating the thermodynamic contribution of configurational entropy. To clarify this, Murty et al. [37] introduced a formal definition of configurational entropy (*S*_config_), as shown in Eq. (1).

1 $${S}_{config}=-R[({\sum }_{i=1}^{N}{x}_{i}{lnx}_{i}{)}_{cation-site}+({\sum }_{j=1}^{M}{x}_{j}{lnx}_{j}{)}_{antion-site}]$$

where *R* is the universal gas constant, and *x*_*i*_, *x*_*j*_ are the molar fractions of each cation and anion species, respectively. Based on the magnitude of *S*_config_, materials are broadly categorized as follows (see Fig. 2a):

- High-entropy materials: *S*_config_≥1.5*R*
- Low-entropy materials: *S*_config_ <1*R*
- Medium-entropy materials: 1.5*R*>*S*_config_≥1*R*

Expanding on this classification, Brahlek et al. [38] proposed a broader framework that incorporates not only entropy values but also the mechanisms driving phase stability. They introduced three distinct material classes (Fig. 2b):

- Compositionally complex materials (CCMs): materials containing multiple elements in one or more sublattices, where phase stability may be governed by kinetic constraints or intrinsic crystallographic stability, rather than entropy.
- HEOs: materials with *S*_config_≥1.5*R*, where entropy makes a meaningful contribution to phase stability.
- ESOs: a subset of HEOs where entropy is the dominant term in the free energy, explicitly stabilizing the single-phase structure.

This classification scheme establishes a rigorous conceptual foundation, clarifying the terminology often used interchangeably in the literature and providing a more unified framework for future HEO research.

While ESOs are technically a distinct subclass of HEOs—defined by their entropy-driven phase stability—for clarity and consistency, this review adopts “HEO” as a general term to refer to all multi-component oxide systems with high configurational entropy.

### Structural Characteristics and Phase Stability Mechanisms of HEOs

As a distinctive subset of high-entropy materials, HEOs possess unique structural and thermodynamic characteristics that underpin their functional versatility. The dynamic interplay between configurational entropy and localized chemical disorder governs the crystallographic evolution of HEOs, modulates defect transport behavior, and supports the stabilization of complex single-phase systems. These mechanisms are particularly relevant for thin-film fabrication and the realization of reliable, tunable resistive switching behavior in memristive devices.

#### Crystal Structures and Phase Stability

**Fig. 3:**
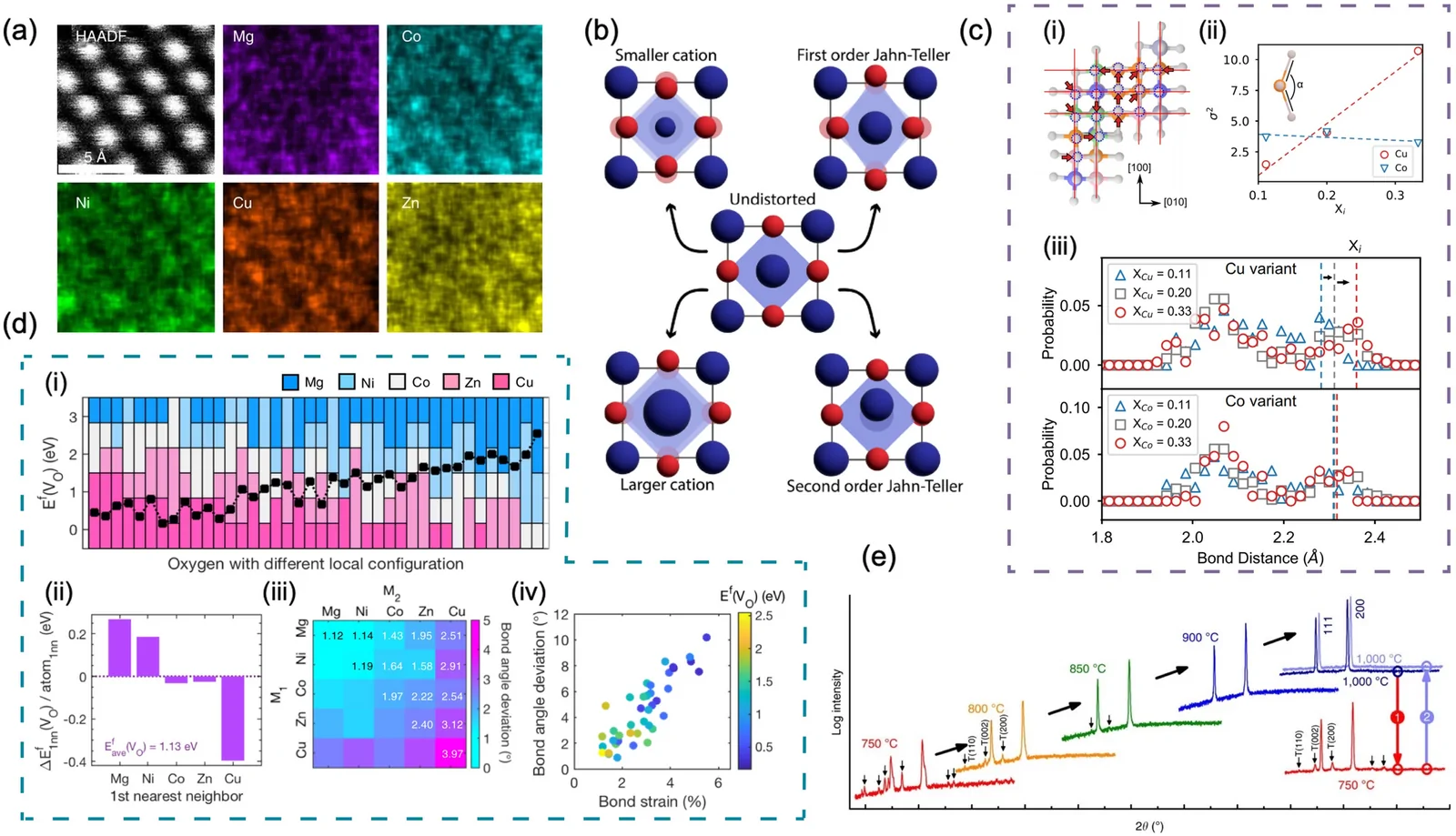
Multiscale structural features and phase stability mechanisms of HEOs. **a** HAADF-STEM image and elemental EDS maps of the (Co_0.2_Cu_0.2_Mg_0.2_Ni_0.2_Zn_0.2_)O system. Reproduced with permission from Ref. [34], licensed under CC BY 4.0. **b** Schematic illustration of cation-induced lattice distortions, including isotropic contraction/expansion, first-order Jahn–Teller distortions, and second-order off-centering. Reprinted with permission from Ref. [29]. Copyright 2023, American Chemical Society. **c** DFT analysis of Cu/Co-rich (Mg, Co, Cu, Ni, Zn)O variants: (i) Relaxed supercell showing Cu-induced distortions and oxygen displacements; (ii) Cu–O and Co–O bond length distributions, revealing Jahn–Teller double peaks for Cu^2+^; (iii) Increasing bond angle variance (*σ*^2^) with Cu content, indicating enhanced local disorder Reprinted figure with permission from ref. [63]. Copyright 2019, American Physical Society. **d** Thermodynamic and structural descriptors of oxygen-vacancy (VO) formation in (Mg, Ni, Co, Cu, Zn)O: (i) VO formation energies across 1NN cation environments; (ii) Element-specific impact on VO energetics; (iii) Correlation with bond-angle deviation; (iv) Linear relation between VO formation energy and local bond strain Reproduced with permission from Ref. [64], licensed under CC BY 4.0. **e** XRD patterns at various annealing temperatures (750–1000 °C), illustrating entropy-driven phase evolution from multiphase to single-phase structure. Reproduced with permission from Ref. [34], licensed under CC BY 4.0

The first successful synthesis of a high-entropy oxide was reported by Rost et al. in 2015 [2], who synthesized a single-phase rock-salt compound, (Co_0.2_Cu_0.2_Mg_0.2_Ni_0.2_Zn_0.2_) O, characterized by high crystallinity, homogeneous elemental distribution, and the absence of secondary phases (Fig. 3a). This seminal study extended the high-entropy design paradigm from metallic alloys to oxides, leading to the exploration of HEOs with a variety of crystal frameworks, including perovskite [39, 40], spinel [41, 42], fluorite [43, 44], and pyrochlore [45, 46] lattices.

Stabilizing a single-phase HEO requires a coordinated balance between entropy-driven thermodynamic forces and crystallographic compatibility. While high configurational entropy effectively lowers the Gibbs free energy and inhibits phase segregation, structural stability is additionally influenced by the ionic radius mismatch, differences in electronegativity, and the tolerance factor specific to the host lattice [47, 48]. Synthesis conditions are equally critical: phase purity and homogeneity can be enhanced through high-temperature annealing (typically between 1150 and 1400 °C) or via nonequilibrium processing routes such as mechanochemical synthesis, which facilitates atomic-scale mixing [49, 50].

Different structural archetypes impart HEOs with distinct physicochemical properties. Rock-salt systems exhibit excellent defect tolerance and thermal resilience, making them ideal for stable conduction pathways [28, 51]. Spinel-type HEOs possess intrinsic ionic/electronic conduction channels, lending themselves to applications in catalysis and energy storage [52, 53]. Perovskites offer highly tunable A/B-site cation substitution, enabling control over magnetism, dielectric response, and transport behavior [54–56]. These structure–property relationships form the foundation for designing reliable and adaptable HEO-based memristive devices.

#### Local Disorder and Its Link to Functional Properties

A defining feature of HEOs is their ability to retain long-range crystalline order while accommodating significant local chemical disorder. At the atomic scale, the coexistence of diverse cation species leads to fluctuations in charge density, orbital occupancy, and lattice strain. These variations drive a variety of local distortions in coordination geometry, including isotropic contraction caused by smaller cations, expansion induced by larger cations, first-order Jahn–Teller distortions, and second-order Jahn–Teller off-centering (Fig. 3b). Together, these distortions generate a structurally complex energy landscape that underpins many of the functional responses observed in HEO-based devices [28, 57, 58].

A representative example is the rock-salt-type (Mg, Co, Cu, Zn, Ni) O system, in which Cu^2+^ ions induce Jahn–Teller distortions within Cu–O octahedra. Density functional theory (DFT) calculations show that approximately 10% of Cu^2+^ sites undergo non-classical axial compressions, breaking local symmetry and modifying charge distribution [59]. This distortion is consistent with previous findings that Cu^2+^ ions promote strong tetragonal elongation in the octahedral environment, leading to four short and two long Cu–O bonds, which destabilize the local lattice symmetry [60]. Concurrently, subtle displacements of anions—often in response to local stress relaxation—introduce additional variations in coordination environments and modulate the surrounding electrostatic potential field [61, 62].

These behaviors are clearly visualized in the DFT-relaxed structural models, which display oxygen displacements, bimodal Cu–O bond length distributions, and Cu-dependent increases in bond angle variance (Fig. 3c). Importantly, these distortions are not isolated perturbations but rather intrinsic features of the entropically stabilized lattice. Additional DFT calculations further demonstrate that oxygen-vacancy formation energies vary considerably across different local cation environments in (Mg, Ni, Co, Cu, Zn) O, ranging from ~ 1.2 to 2.4 eV depending on the first-nearest-neighbor configuration (Fig. 3d (i)). Cu-rich sites tend to lower the formation energy, whereas Mg and Ni environments raise it (Fig. 3d(ii)). This energetic diversity correlates strongly with bond-angle deviation and local strain, which serve as effective structural descriptors of defect formation (Fig. 3d(iii, iv)). As discussed by Kotsonis et al. [28], the inherent configurational disorder in HEOs softens the lattice and promotes local symmetry breaking, thereby enabling metastable or distorted structural motifs that contribute to functional adaptability.

This structural plasticity is not merely a crystallographic curiosity—it plays an active role in defining material performance. Local disorder improves defect tolerance, stabilizes intermediate resistance states, and enables adaptable charge transport. In memristive devices, the coexistence of ordered and disordered domains facilitates the formation of multiple, spatially distributed conduction pathways, which improves device uniformity and suppresses filament overgrowth. Moreover, such configurational diversity provides the structural basis for analog conductance modulation and synaptic-like plasticity behavior, as explored in Sect. 6.3.

Finally, it should be noted that conventional X-ray diffraction techniques may fail to resolve the extent of local disorder, as they primarily capture long-range average symmetry. To uncover short-range structural deviations and local heterogeneity, advanced tools such as total scattering and pair distribution function (PDF) analysis are essential [28]. These techniques enable a more faithful reconstruction of the structural complexity inherent in high-entropy systems, which is critical to understanding and engineering their emergent functional behaviors.

#### Entropy-Driven Phase Evolution and Thermal Stability

The thermodynamic foundation for phase stability in HEOs is governed by the classical Gibbs free energy relation. The thermodynamic basis for phase stability in HEOs stems from the classical expression of Gibbs free energy changes (Δ*G*_mix_) as defined in Eq. (2).

2 $$\Delta {G}_{mix}=\Delta {H}_{mix}-T\Delta {S}_{mix}$$

where Δ*H*_mix_ is the enthalpy, Δ*S*_mix_ is the configurational entropy, and T is temperature. At elevated temperatures, a sufficiently large entropy contribution (–*T*Δ*S*_mix_) can outweigh enthalpic penalties, stabilizing single-phase solid solutions and suppressing phase segregation [34, 60, 65].

Experimental observations confirm that HEOs undergo a transformation from multiphase mixtures to single-phase structures upon high-temperature annealing (above 900 °C, Fig. 3d), whereas the reverse transition may occur during cooling if the entropic stabilization is no longer sufficient. Notably, thermodynamic simulations have shown that five-component systems achieve entropy stabilization at significantly lower temperatures than four-component ones, reinforcing the critical role of cation diversity in suppressing phase separation [60]. Interestingly, defect states such as oxygen vacancies have been found to locally enhance entropy, improving mid-temperature structural stability [66].

Although lattice distortions and cation-size mismatch can introduce destabilizing perturbations, moderate mismatch can in fact increase configurational diversity and deepen the entropy potential well, thus enhancing phase robustness [60, 67].

Computational studies using DFT and Monte Carlo simulations have revealed temperature- and composition-dependent profiles of Gibbs free energy in multi-cation systems, offering theoretical validation for entropy-dominated phase behavior [68]. In situ synchrotron X-ray diffraction has further confirmed the persistence of random cation distributions and the suppression of unwanted secondary phases under thermal and mechanical stress.

This unique combination of high-temperature stability and defect-mediated tunability provides a critical physical safeguard for HEO-based memristors—ensuring reliable operation, thermal resilience, and long-term state retention under real-world conditions.

### Synthesis Strategies and Structural Tunability of HEOs

The synthesis route plays a crucial role in determining the structural homogeneity, phase stability, and defect landscape of HEOs, all of which directly influence their functional performance in memristive and other electronic applications. As the field has matured, synthesis strategies have evolved from traditional thermodynamic approaches toward more controllable and structurally tunable methods. These advances allow precise regulation of composition, microstructure, and scalability. Broadly, synthesis routes can be categorized into two classes: conventional equilibrium-based techniques and emerging rapid or nonequilibrium processes.

#### Conventional Equilibrium Synthesis Methods

Thermal equilibrium-based synthesis remains the most widely adopted approach for HEO fabrication. These methods typically rely on high-temperature treatments to enable multi-cation diffusion and lattice reorganization, leading to the formation of thermodynamically stable, single-phase oxides. Common strategies include solid-state synthesis, sol–gel processing, and co-precipitation.

Among them, solid-state synthesis is widely used due to its simplicity and scalability. It involves mixing metal oxides or carbonates—often by ball milling—followed by calcination at elevated temperatures (typically above 900 °C) to promote crystallization and phase homogenization [69, 70]. This method has been applied to various HEO structure types, including rock-salt, spinel, and perovskite oxides [34, 71, 72].

However, prolonged high-temperature calcination, a hallmark of conventional solid-state synthesis, imposes several practical limitations, particularly for thin-film device applications. In situ transmission electron microscopy (TEM) studies of (Cr, Mn, Fe, Co, Ni)_3_O_4_ reveal that element incorporation occurs sequentially (e.g., Ni/Mn at ~ 500 °C, Co at ~ 600 °C, and Fe/Cr above 700 °C), leading to extended diffusion times and delayed lattice homogenization [70]. Moreover, severe grain coarsening (~ 77 nm) and local compositional inhomogeneity were observed after 12 h of calcination, which are detrimental to film uniformity, device-to-device consistency, and long-term electrical reliability. These effects highlight the critical challenge of maintaining nanoscale compositional control under high-temperature conditions—an issue of particular concern for industrial integration and large-area film deposition.

The sol–gel method uses molecular-level metal–organic complexation followed by hydrolysis and condensation to form a homogeneous gel. Subsequent heat treatment yields oxide phases with high compositional uniformity [46]. This method offers fine control over morphology, surface area, and porosity, making it especially suitable for high-surface-area powders and thin films [73]. Notably, Gupta et al. showed that an inverse-spinel (Cu, Ni, Zn, Al, Fe)_3_O_4_ HEO can be obtained via a self-combustion sol–gel process at only 350 °C for 1 h, producing ≈ 8 nm porous flakes in kilogram-scale batches and thereby reducing the thermal budget by nearly an order of magnitude compared with conventional furnace cycles [74]. Although pore collapse may occur during thermal treatment, adjusting parameters such as pH, solvent system, and annealing protocol can significantly enhance film quality and crystallinity [75–77].

In co-precipitation synthesis, multiple metal ions are simultaneously precipitated—typically as hydroxides or carbonates—and subsequently calcined to form homogeneously mixed HEOs [78–80]. This approach allows nanoscale control over particle size and composition. Challenges such as agglomeration and surface area loss are common, but can be mitigated using dispersants or optimized post-treatment conditions [81, 82].

#### Emerging Rapid and Nonequilibrium Synthesis Techniques

To overcome the time- and energy-intensive nature of traditional thermal methods, several rapid and nonequilibrium synthesis techniques have recently been applied to HEOs. These methods offer faster reaction kinetics, finer nanostructural control, and enhanced defect engineering capabilities:

- **Joule Heating**
  Uses resistive heating to trigger ultrafast crystallization (within seconds), reducing grain growth and preserving nanoscale features. This technique is ideal for high-throughput film screening and rapid prototyping [69, 83].
- **Carbon Thermal Shock (CTS)**
  Applies millisecond-scale thermal pulses to induce instantaneous crystallization. The rapid heating–cooling cycle improves phase purity and suppresses unwanted secondary phases [84].
- **Low-Temperature Plasma Processing**
  Enables the synthesis of defect-rich nanostructures under mild thermal conditions. This technique is effective for tuning surface chemistry, oxygen vacancy content, and catalytic activity in multifunctional HEOs [85].
- **Mechanochemical Synthesis**
  A solvent-free, high-energy ball milling approach that promotes atomic-level mixing and amorphization, enabling oxide formation without calcination. It is energy-efficient, scalable, and suitable for complex compositions [86].

Compared to conventional high-temperature sintering, these nonequilibrium methods offer reduced energy consumption, enhanced film quality, and better control over switching-relevant features such as defect landscapes and grain boundaries. Importantly, their scalability, material throughput, and compatibility with existing deposition platforms make them promising candidates for integrating HEO memristors into practical neuromorphic hardware.

### Multiphysical Properties of HEOs

HEOs, by virtue of their compositional complexity and entropy-stabilized structural frameworks, exhibit outstanding performance across multiple physical domains. In addition to tunable electrical, thermal, and optical properties, certain HEO systems also exhibit magnetic ordering due to multi-cation interactions, offering additional functional flexibility. These cross-disciplinary advantages not only expand their application potential in energy systems and catalysis but also lay a robust functional foundation for memristive devices, where controlled switching dynamics and synaptic behaviors are essential.

#### Electrochemical Performance

The synergistic distribution of multiple cation species and the associated defect homogenization in HEOs enhance structural robustness and ionic mobility, both of which are essential for field-driven conduction in memristive systems. In energy-related applications, such as lithium- and sodium-ion batteries, HEOs demonstrate long-term cycling durability by suppressing phase separation and alleviating lattice strain accumulation [87, 88]. Spinel-type HEOs like (Cu, Ni, Fe, Mn, Co)_3_O_4_, provide interconnected ionic and electronic transport pathways, maintaining high capacitance even under high-rate charge–discharge conditions [74].

**Fig. 4:**
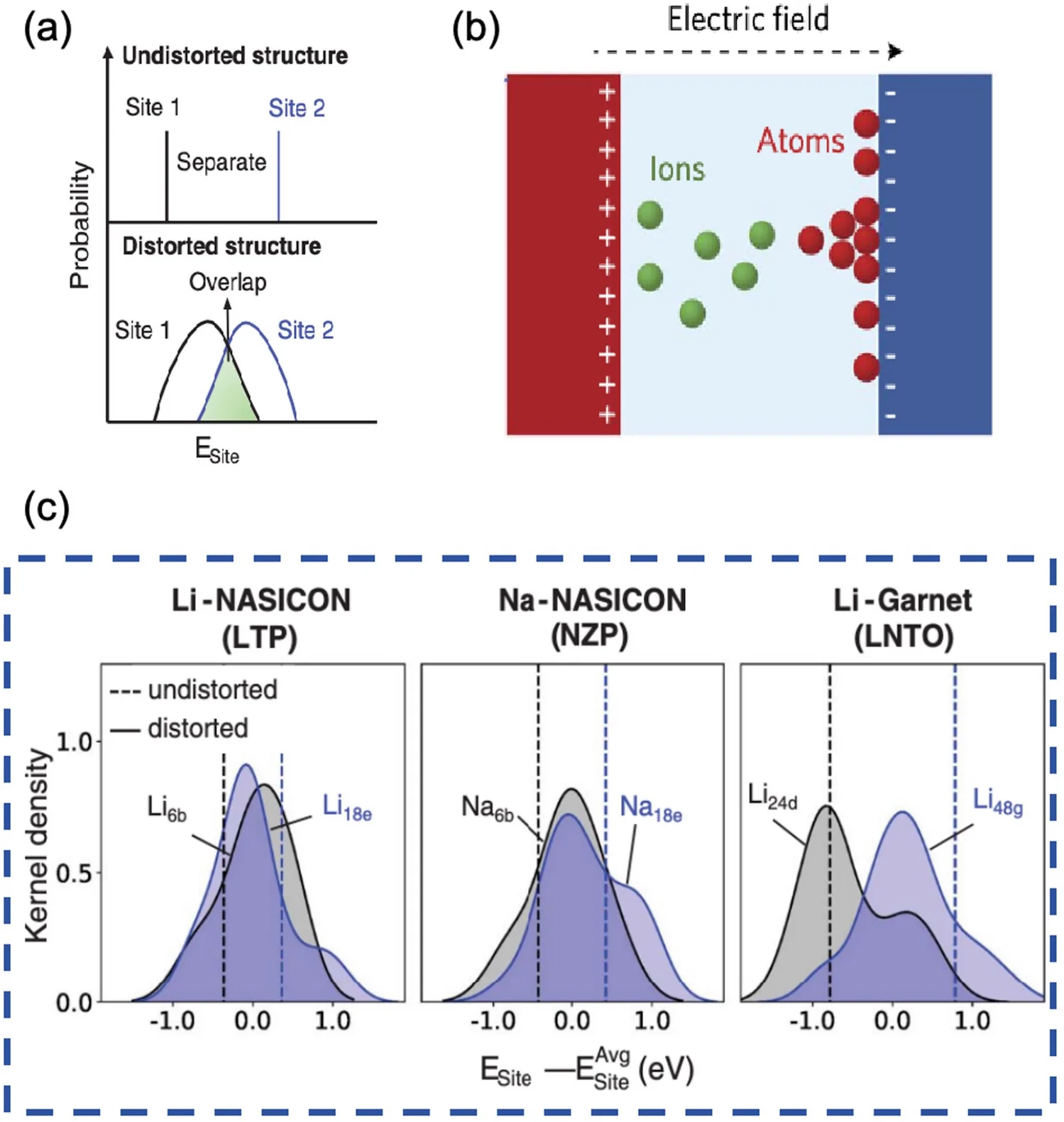
Electrochemical characteristics and ion migration mechanisms in HEOs. **a** Energy level distributions at different cation sites in undistorted and distorted lattice configurations. Reproduced with permission from Ref. [89], licensed under CC BY 4.0. **b** Schematic illustration of ion and atom dynamics under an applied electric field. Reproduced with permission from Ref. [92], Copyright 2018, Springer Nature. **c** Kernel density plots of site energy distributions in representative HEO-based structures, including Li-NASICON, Na-NASICON, and Li-Garnet, comparing distorted and undistorted environments. Reproduced with permission from Ref. [89], licensed under CC BY 4.0

From a resistive switching perspective, entropy-driven lattice disorder and multi-cation dynamics significantly reduce energy barriers for ion migration. Zeng et al. [89] demonstrated that local energy level broadening in disordered HEO frameworks facilitates ultrafast ionic conduction, which is critical for designing defect-tolerant and energy-efficient switching media. Local structural distortions induce overlapping energy distributions between adjacent ionic sites, effectively smoothing migration pathways and enhancing ion percolation (Fig. 4a).

In this context, the migration of ions and atoms under an electric field is critical for understanding the conduction mechanisms in HEO-based devices. Ionic motion is typically decoupled from the slower drift of atoms, leading to field-induced redistribution of species and the formation of conductive filaments or pathways (Fig. 4b). The structural dynamics under electric fields are thus a key element in the switching process and performance stability.

The energy distributions for Li-NASICON, Na-NASICON, and Li-Garnet materials at different cation sites further highlight the influence of structural distortion on ionic behavior. Kernel density analysis reveals that distorted lattices exhibit broader site energy distributions, facilitating smoother ion migration compared to undistorted structures (Fig. 4c). These results validate the role of configurational entropy in boosting ionic mobility and provide a mechanistic foundation for engineering fast-switching media in HEO-based memristive devices.

Beyond bulk ionic conduction, the multi-cation environment and high density of oxygen vacancies in HEOs also activate interfacial processes such as the oxygen evolution reaction (OER) and oxygen reduction reaction (ORR) [90, 91]. These same features—enhanced electron transport, lattice distortion, and abundant reaction sites—translate effectively into memristive functions, where interface-driven conduction, oxygen vacancy mobility, and ion–electron interactions are pivotal for device performance.

#### Thermophysical Properties

The large mass variance and lattice distortion introduced by multi-element incorporation result in enhanced phonon scattering in HEOs, leading to ultralow thermal conductivity and high thermal stability [46, 93]. This scattering arises from both mass disorder and local strain-field fluctuations, which collectively disrupt phonon coherence across multiple length scales. Notably, bond-length variations and heterogeneous elastic fields further amplify this effect, suppressing thermal transport more effectively.

**Fig. 5:**
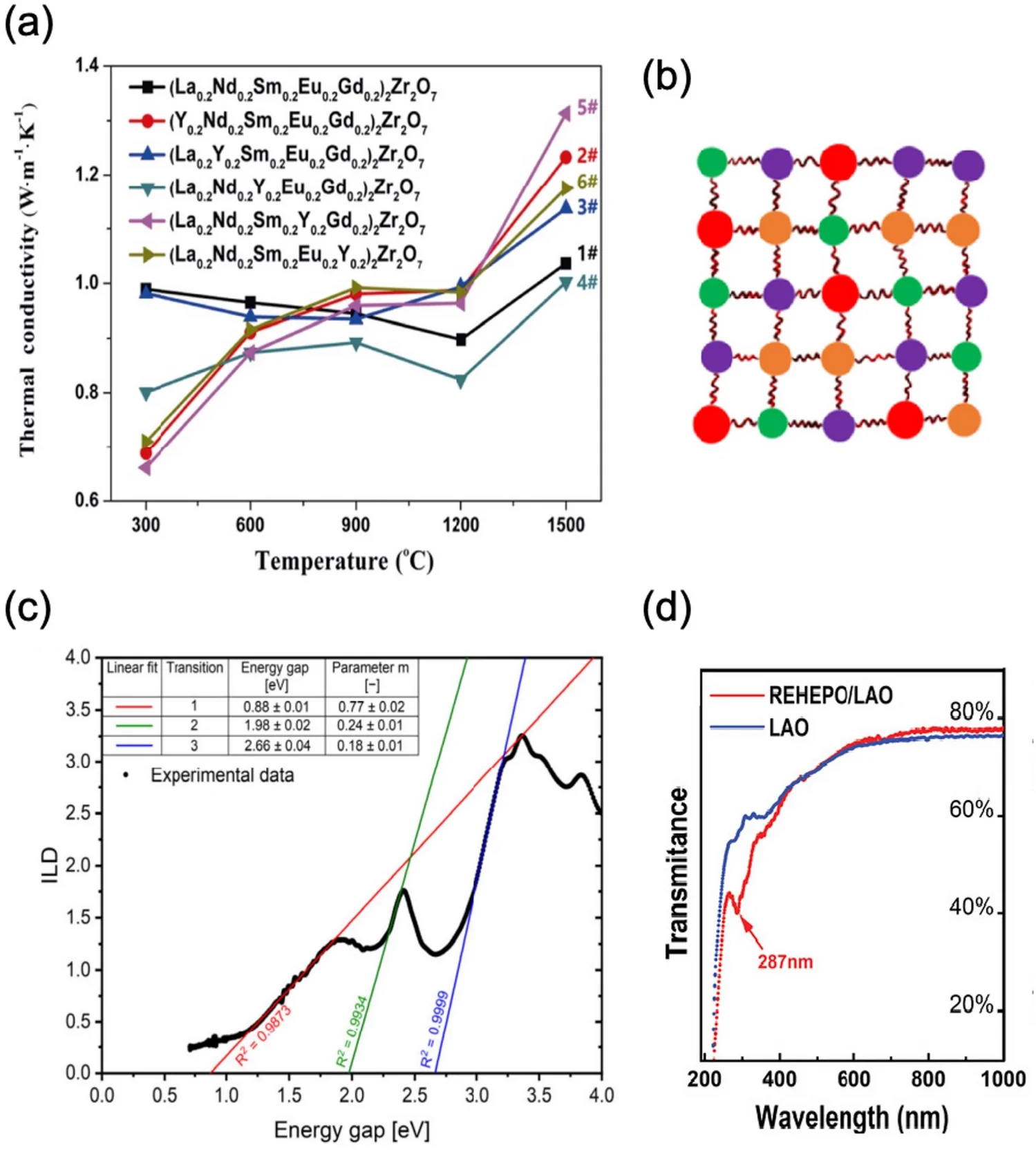
Thermal and optical properties of HEOs. **a** Thermal conductivity of various rare-earth-based high-entropy zirconates over 300–1500 °C, demonstrating ultralow *κ* values in the range of 0.7–1.2 W m^−1^ K^−1^. Reproduced with permission from Ref. [46], licensed under CC BY 4.0. **b** Schematic representation of phonon scattering in high-entropy lattices caused by mass disorder and interatomic force constant fluctuations, which disrupt coherent phonon propagation. Reproduced with permission from Ref. [94]. Copyright 2024, Elsevier. **c** Tauc analysis of REHEPO thin films showing multiple electronic transitions with fitted optical bandgaps at 0.88, 1.98, and 2.66 eV. Reproduced with permission from Ref. [95], licensed under CC BY 4.0. **d** UV–Vis transmittance spectra of REHEPO/LAO and LAO, indicating strong absorption near 287 nm due to entropy-modulated electronic structures. Reproduced with permission from Ref. [96], licensed under CC BY 4.0

Experimental studies on high-entropy pyrochlores have confirmed the persistence of ultralow thermal conductivity across a broad temperature range, supporting the critical role of configurational disorder in suppressing phonon transport (Fig. 5a). The thermal conductivity remains remarkably low (0.7–1.2 W m^−1^ K^−1^) from 300 to 1500 K, significantly lower than that of conventional oxides such as YSZ or Al_2_O_3_ (typically > 10 W m^−1^ K^−1^) [46]. This reduction in thermal conductivity can be attributed to the phonon scattering mechanisms illustrated in Fig. 5b, where the random distribution of cations and lattice distortions create additional scattering centers, thereby restricting phonon mean free paths (MFP) and enhancing phonon localization [94].

In addition to entropy-driven thermodynamic stabilization, local structural distortions, particularly those induced by Jahn–Teller active cations like Cu^2+^, introduce tensile stress and increase bond-length variance. These distortions introduce tensile stresses and increase bond-length variance, creating more scattering sites that disrupt phonon transport. The resulting increase in scattering centers contributes to the ultralow thermal conductivity observed in HEOs.

Moreover, certain HEOs exhibit outstanding thermal robustness, maintaining single-phase structural integrity even at temperatures exceeding 1120 K [60]. This combination of ultralow thermal conductivity and high-temperature phase stability ensures reliable functionality of HEO-based memristive devices under high-field, high-frequency, and harsh environmental operating conditions.

#### Optoelectronic Properties

HEOs possess tunable bandgap energies and efficient broadband light absorption, positioning them as promising candidates for applications in solar energy conversion, photodetection, and optoelectronic neuromorphic systems [97–99]. Through bandgap engineering and controlled defect-state modulation, memristive devices can be designed with photo- and thermal responsiveness, enabling their operation under optical and thermal stimuli.

Particularly, rare-earth-based perovskite HEOs have demonstrated promising optoelectronic behaviors due to their compositional complexity and defect engineering potential. For instance, a high-entropy (Gd, Nd, La, Sm, Y)CoO_3_(RECO) thin film exhibited a direct allowed electronic transition with a tunable optical bandgap of approximately 0.88 eV and broad UV–Vis-NIR absorption (Fig. 5c). Similarly, a (La_0.2_Lu_0.2_Y_0.2_Gd_0.2_Ce_0.2_)AlO_3_ high-entropy perovskite oxide thin film showed high optical transparency (65%–78%) across the visible to near-infrared range and a direct bandgap of about 5.52 eV (Fig. 5d).

The optoelectronic tunability of HEOs arises from entropy-driven modulation of oxygen vacancy distributions, cationic site disorder, and multivalent ion configurations, which collectively reshape the local electronic landscape. These features enable light-enhanced resistive switching, persistent photoconductivity, and thermally assisted conductance modulation—key behaviors for designing multimodal memristive systems.

Such multimodal responsiveness lays a critical foundation for vision-inspired and temperature-adaptive neuromorphic applications, where dynamic regulation of synaptic weights under electrical, optical, and thermal stimuli becomes achievable.

## Summary

HEOs represent a versatile class of materials whose configurational entropy and multi-cation complexity enable unique structural, thermal, and electronic functionalities. From entropy-stabilized crystal frameworks and tunable phase behavior to rich local disorder and multiphysical coupling, the intrinsic characteristics of HEOs offer an unprecedented platform for device-level integration.

Across the structural dimension, the coexistence of long-range order and short-range distortion promotes defect tolerance, while phase stability mechanisms driven by entropy offer resilience under thermal and electrical stress. In terms of synthesis, the evolving toolkit of both equilibrium and nonequilibrium methods has expanded the compositional space of HEOs, enabling precise control over morphology, phase purity, and functional properties.

Functionally, HEOs exhibit high ionic mobility, thermal insulation, magnetic adjustability, and photoresponsivity—all of which are desirable for next-generation memristive devices. Their ability to host diverse defect chemistries and undergo field-responsive transitions lays a strong foundation for resistive switching, multilevel storage, and neuromorphic plasticity.

In sum, HEOs bridge the gap between complex materials design and adaptive device behavior. Their compositional tunability and emergent functionalities position them not only as promising candidates for memory and logic devices but also as a transformative material platform for brain-inspired hardware systems.

## Device Mechanisms and Material Innovations in High-Entropy Oxide Memristors

### Operating Principles and Switching Characteristics of Memristors

The memristor, first postulated by Chua [7] as a fundamental passive circuit element linking charge and magnetic flux (Fig. 5a), introduced a new paradigm for resistance modulation rooted in historical charge flow [100]. In practical devices, this phenomenon is manifested as resistive switching (RS) behavior, wherein external electrical stimuli induce reversible transitions between a high-resistance state (HRS) and a low-resistance state (LRS), with the resistance state retained even after power removal—thus enabling non-volatile memory operation [101, 102]. This history-dependent modulation forms the physical basis for information storage and learning processes in memristive systems.

At the microscopic level, RS behavior arises from a variety of electrochemical processes, including ion migration, defect redistribution, charge trapping, and phase transitions within the active material [27]. These mechanisms dynamically modulate conductive pathways or interface barriers, enabling structure–state–function coupling in memristive systems.

**Fig. 6:**
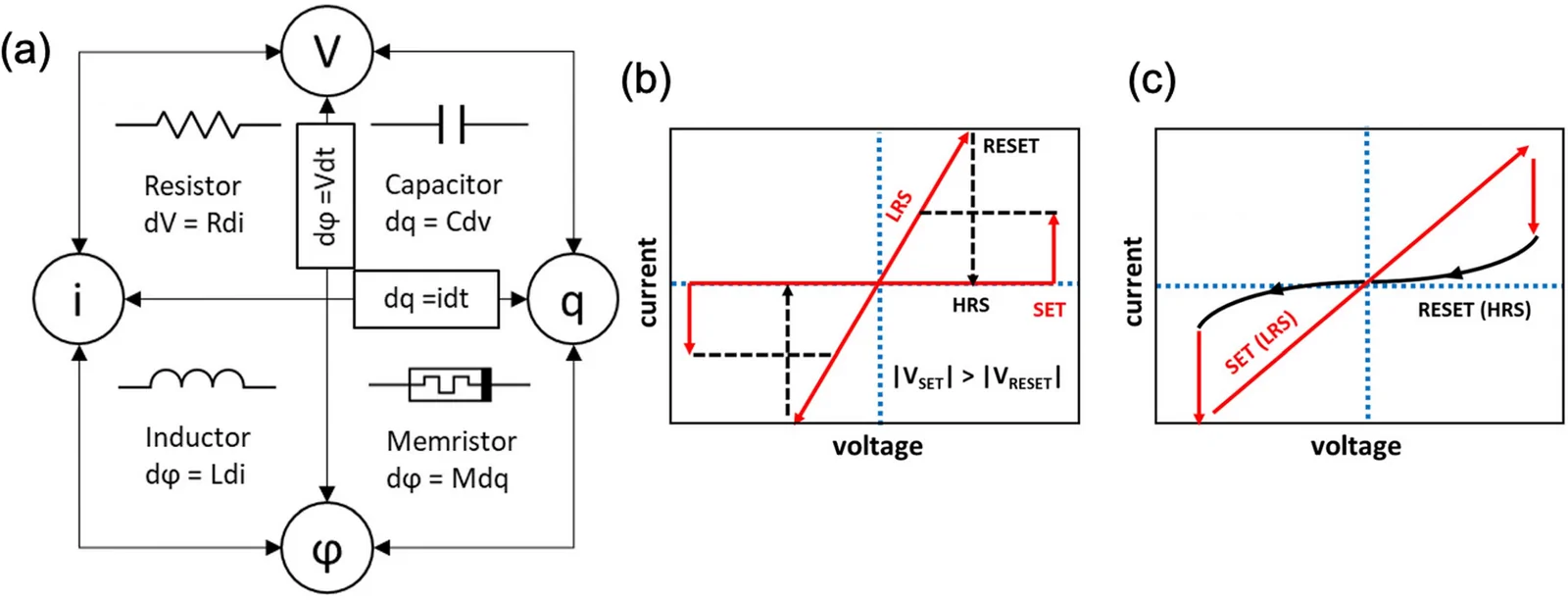
Fundamental definition and characteristic switching modes of memristors. **a** Schematic of the memristor as the fourth fundamental passive circuit element, defined by the relationship between charge and magnetic flux, complementing the resistor, capacitor, and inductor in circuit theory.** b** Unipolar resistive switching, where both SET and RESET processes occur under the same voltage polarity, with the SET voltage typically higher than the RESET voltage. **c** Bipolar resistive switching, where the SET and RESET transitions occur under opposite voltage polarities. Reproduced with permission from Ref. [107], licensed under CC BY 4.0
- Bipolar switching, where SET and RESET processes require opposite voltage polarities, often associated with field-driven ion migration or valence change mechanisms, commonly seen in TiO_2_ and HfO_2_ [105, 106] (Fig. 6c).

Based on voltage polarity dependence, RS behavior can be broadly categorized into two modes:

- Unipolar switching, where both SET (HRS → LRS) and RESET (LRS → HRS) transitions occur under the same voltage polarity, primarily driven by voltage amplitude and Joule heating effects, as typically observed in binary oxides such as NiO and ZnO [103, 104] (Fig. 6b);

While the macroscopic switching modes are readily distinguishable through electrical measurements, the underlying physical mechanisms—such as ion migration, defect evolution, and phase transformations—play critical roles in dictating switching dynamics, variability, and functional scalability. These microscopic processes are discussed in detail in the following section.

### Microscopic Mechanisms and Classification of Resistive Switching

In conventional oxide-based memristors, resistive switching behaviors are predominantly governed by three microscopic mechanisms: valence change mechanisms (VCM), electrochemical metallization mechanisms (ECM), and thermochemical mechanisms (TCM) [108, 109]. Each mechanism reflects a distinct physical pathway for conductance modulation and critically influences device performance attributes such as switching polarity, energy consumption, endurance, and retention.

**Fig. 7:**
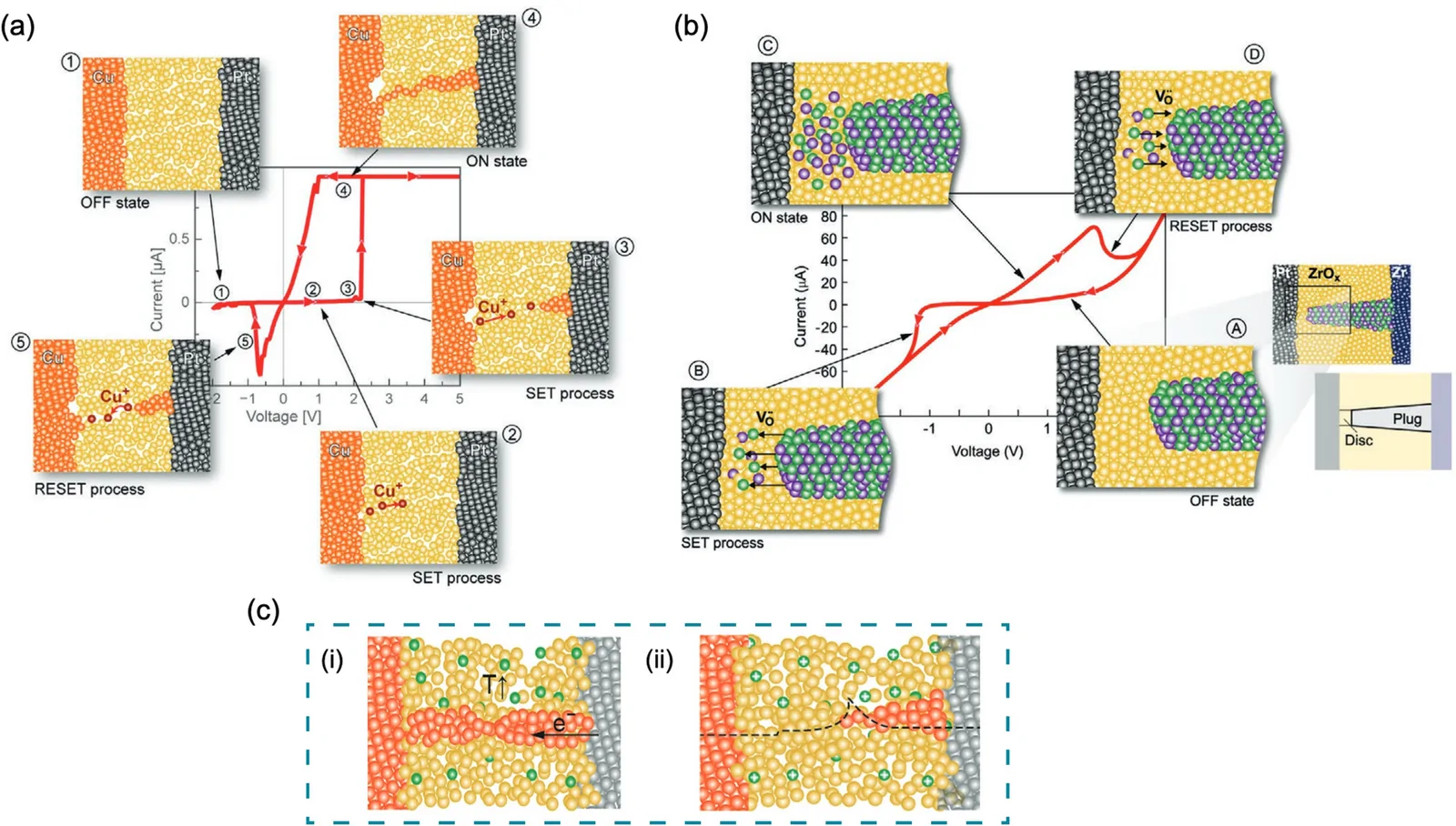
Schematic comparison of three resistive switching mechanisms. **a** ECM mechanism: field-driven migration of active metal cations from the top electrode through the dielectric and their subsequent reduction near the bottom electrode, resulting in the formation and rupture of metallic filaments. **b** VCM mechanism: redistribution of oxygen vacancies under electric field, modifying the local valence states of cations and enabling the formation of conductive channels. **c** TCM mechanism: local Joule heating initiates filament formation (i), while thermal and surface tension effects such as the Gibbs–Thomson instability lead to filament thinning and eventual rupture (ii). Reproduced with permission from Ref. [116], licensed under CC BY 4.0

VCM-based switching, widely observed in transition-metal oxides such as TiO_2_ and HfO_2_, involves electric-field-driven migration of oxygen vacancies (*V*_O_) and associated valence changes of metal cations. Conductive filaments formed by *V*_O_ accumulation enable low-resistance pathways, which can be reversibly ruptured to restore high-resistance states [110, 111]. VCM devices typically exhibit bipolar switching behavior, good endurance, and analog conductance tunability; however, they often require a high-voltage forming step and suffer from stochastic filament formation, leading to variability in switching parameters. These switching dynamics are conceptually illustrated in Fig. 7a.

ECM-based switching, in contrast, relies on the field-driven migration of active metal cations (e.g., Ag^+^, Cu^+^) originating from an electrochemically active electrode [112, 113]. These cations migrate through the dielectric and are reduced near the inert electrode, forming metallic filaments that bridge the electrodes. ECM devices are notable for their ultra-low switching energy and high-speed operation, yet they frequently encounter challenges related to filament volatility and poor data retention stability. These switching dynamics are as visualized in (Fig. 7b).

TCM-based switching mechanisms are driven by local Joule heating effects, which induce thermal-assisted phase transformations or defect redistributions within the switching medium [114, 115]. Such processes can facilitate fast switching and simple device structures but are often associated with unipolar switching behavior, lower endurance, and higher energy consumption compared to VCM and ECM systems. The thermally driven evolution and breakdown of conductive paths are shown in Fig. 7c(i–ii).

While these microscopic mechanisms have enabled substantial advancements in resistive switching devices, they also inherently impose limitations—including stochastic filament formation, forming requirements, thermal degradation, and limited scalability for multilevel conductance modulation. Addressing these persistent challenges necessitates the exploration of novel material platforms with improved defect stability, controllable switching dynamics, and intrinsic resistance to variability. In this context, HEOs have recently emerged as promising candidates, offering unique opportunities to overcome the bottlenecks inherent in conventional systems. Their roles and potential challenges in memristive switching are discussed in Sect. 4.4.

### Comparative Analysis of Traditional and High-Entropy Oxide Memristors

Memristive devices commonly adopt a metal–insulator–metal (MIM) architecture, in which device performance primarily depends on the active RS layer, while electrode materials and interface engineering significantly influence conductive filament formation and switching dynamics. Extensive research has explored four principal material classes: binary oxides, perovskites, 2D materials, and organic polymers. This section systematically compares these traditional material systems against emerging HEOs, highlighting critical advantages of the latter across seven performance criteria essential for neuromorphic hardware—Forming-Free, ON/OFF Ratio, Endurance, Retention, Switching Speed/Energy, Multilevel States, and CMOS Compatibility (Table 1).。

**Table 1:** Performance metrics for traditional and high-entropy oxide memristors

Material system	Representative material	Forming-free	ON/OFF ratio	Endurance	Retention	Switching speed/energy	Multilevel states	CMOS compatibility CMOS	Refs.
Binary-oxides	HfO_2_	No	≈10^3^–10^4^	1.2 × 10^11^	3 × 10^8^ s (ext.)	5 ns/–	24	Yes	[117]
	TiO_2_-C oO	Yes	~ 10^3^	1 × 10^3^	1 × 10^4^ s	–/0.4 V, 2.5 µA (~ 1 µW)	✓ (via *V*_stop_)	Yes	[118]
Perovskites	MAPbI_3_ quantum wires	Yes	1 × 10^7^	6 × 10^6^	> 2 years (ext.)	~ 100 ps/–	4	Limited	[119]
2D Materials	MoS_2_	No	~ 10^5^	–	10^4^ s	ns/–	5	Limited	[120]
Organic polymers	Hetero-electrolyte polymer	No	1 × 10^3^	2 × 10^2^	–	–	–	Limited	[121]
HEOs	(Zr, Hf, Nb, Ta, Mo, W)_2_O_5−*x*_	Yes	6–15	7 × 10^4^	7.2 × 10^4^ s	–	64	Yes	[33]
	(Cr, Mn, Fe, Co, Ni)_3_O_4_	Yes	1 × 10^5^	4.5 × 10^3^	> 10^4^ s	–	32 (analog)	Yes	[32]

Binary oxides, exemplified by hafnium oxide (HfO_2_), have emerged as the mainstream commercial ReRAM materials due to their high dielectric constants and full compatibility with CMOS back-end-of-line (BEOL) processes, enabling sub-nanosecond switching speeds. However, the underlying mechanism relies on randomly forming and rupturing oxygen vacancy filaments within the oxide layer, necessitating a high-voltage electroforming step at device initialization. This randomness induces significant variability both within devices and across cycles, complicating large-scale integration and system reliability. Although program-and-verify schemes can improve the stability of intermediate states, they generally only enable a modest number of reliably distinguishable levels, which still constrains weight precision in neuromorphic applications [117].

To address these variability and power consumption issues associated with binary oxides, phase-separated oxides such as the TiO_2_–CoO system have been explored. This system employs tailored microstructural interfaces that achieve intrinsic forming-free switching at significantly reduced voltages (around 0.4 V) and current levels (µA-range), substantially reducing device variability (σ/μ ≈ 3.6%) and power consumption [118]. Nonetheless, these materials exhibit moderate ON/OFF ratios and relatively limited endurance. Additionally, their long-term stability and performance under elevated temperatures or flexible substrates remain insufficiently validated, highlighting continuing challenges for practical integration.

Perovskite materials, notably MAPbI_3_ quantum wires, have set benchmarks in single-device performance, demonstrating exceptional ON/OFF ratios, ultra-fast switching speeds, impressive endurance, and extrapolated retention periods surpassing two years at room temperature (Table 1). Despite these remarkable performance metrics, their practical implementation is significantly hindered by reliance on sophisticated nanofabrication processes and precision lithography. Furthermore, their susceptibility to environmental factors such as humidity and temperature fluctuations, combined with difficulties in achieving consistent device performance across large arrays, severely limits their scalability and real-world deployment.

2D materials, particularly monolayer chemical vapor deposition (CVD)-grown MoS_2_ devices, require an initial electroforming step at approximately 3 V to activate resistive switching. Once formed, they deliver high ON/OFF ratios and nanosecond switching speeds (Table 1). Multilevel conductance modulation is achieved by tuning the stop-voltage (*V*_stop_), enabling five discrete resistance states. However, these devices suffer from limited cycling endurance (~ 20 cycles), wafer-scale film uniformity issues, and interface defects or cracks introduced during transfer and annealing, all of which hinder robust CMOS-compatible BEOL integration and large-scale array deployment.

Organic polymer-based devices, such as the PMMA-based hetero-electrolyte memristors reported by Park et al., offer advantages of flexibility and low-temperature solution processability (< 105 °C), alongside intrinsic self-selecting characteristics. Nevertheless, these devices still require relatively high electroforming voltages (6–8 V), exhibit limited endurance (~ 200 cycles), and lack stable, discrete multilevel states. Additionally, their retention performance has not been extensively validated for prolonged periods, rendering them insufficient for sustained, high-density storage applications.

In essence, conventional material systems inherently balance one advantage against a significant limitation: binary oxides offer CMOS compatibility but face high variability; perovskites excel in speed but suffer from environmental instability; 2D materials achieve compactness yet struggle with wafer-scale uniformity; polymers are processed at low temperatures but fail to meet endurance or retention requirements.

In contrast, HEOs offer a fundamentally different approach. By incorporating multiple cation species into a single oxide lattice, configurational entropy stabilizes a dense, spatially distributed vacancy network. This network enables forming-free operation at sub-volt biases and significantly reduces device-to-device variability (*σ*/*μ* ≈ 8%; further improvements targeted below 5%). Moreover, the multicomponent nature of HEOs facilitates smooth, linear analog weight tuning across 32–64 discrete states, maintaining nanosecond-scale switching at ultra-low energies (fJ–pJ/bit)—attributes highly desirable for neuromorphic computing. Additionally, HEO films can be easily deposited by low-temperature processes such as sol–gel or self-propagating combustion methods (< 300 °C), seamlessly integrating with BEOL processes and large-area substrates, thus bypassing the lithographic complexity and transfer-related challenges encountered by perovskites and 2D materials.

Overall, HEOs concurrently deliver forming-free operation, notably reduced variability, low-energy nanosecond switching, extensive analog tunability, and comprehensive CMOS BEOL compatibility (Table 1). These advantages uniquely position HEOs to bridge the existing gap between laboratory demonstrations and scalable neuromorphic computing hardware. The following section delves deeper into the Opportunities and Challenges for HEO Memristors.

### Opportunities and Challenges for High-Entropy Oxide Memristors

Building upon the fundamental principles of resistive switching mechanisms, HEOs have recently emerged as promising candidates for next-generation memristive devices. Unlike conventional systems constrained by stochastic filament formation, thermal instability, and limited analog control, HEOs offer a fundamentally distinct approach toward resistive switching, leveraging configurational entropy to stabilize complex defect landscapes and enhance operational reliability [28].

One of the most notable advantages of HEO-based memristors is their propensity for forming-free switching. The multicomponent cationic environment, combined with an intrinsic high density of oxygen vacancies, facilitates spontaneous conduction path activation without requiring an initial electroforming step [32, 33]. This characteristic significantly enhances device-to-device uniformity and reduces operational overhead. Furthermore, the configurational disorder intrinsic to HEOs leads to spatially distributed defect stabilization, suppressing localized hot-spot formation and mitigating filament-driven stochasticity—thus promoting more gradual and controllable resistance modulation [28].

HEOs also exhibit strong potential for multistate, analog conductance control, which is critical for neuromorphic computing applications. The synergistic interplay among multiple cation species creates a diversified local energy landscape, enabling continuous adjustment of conduction pathways in response to external stimuli [33]. Such multilevel modulation behavior mirrors biological synaptic plasticity and is instrumental for realizing high-precision learning dynamics in hardware-implemented neural networks.

Despite these promising attributes, several challenges must be addressed to fully harness the capabilities of HEO-based memristors. The complexity of cationic distributions introduces variability in migration pathways and activation energies, complicating the predictability and reproducibility of switching behaviors [32, 60]. Additionally, the coupling between defect migration, phase transitions, and redox reactions can result in intricate and dynamic switching mechanisms that are difficult to decouple and model accurately. These factors pose significant hurdles for achieving deterministic control over switching thresholds, endurance, and multistate precision.

Moreover, while configurational disorder enhances defect tolerance, it also inherently broadens the statistical distribution of defect states, potentially impacting cycle-to-cycle stability and analog state retention [28, 96]. Developing targeted defect engineering strategies—such as selective cation substitution, controlled vacancy injection, and interface modulation—will be critical to overcoming these limitations.

In summary, HEOs provide a compelling new materials platform for overcoming the intrinsic bottlenecks faced by conventional memristive systems. Their unique combination of entropy-stabilized structures, defect-driven tunability, and intrinsic multistate capability positions them as promising candidates for energy-efficient, reliable, and scalable neuromorphic devices. The following chapter delves deeper into the device architectures, switching characteristics, and synaptic functionalities enabled by HEO-based memristors.

## Summary and Outlook

This chapter has reviewed the fundamental resistive switching principles, the classification of microscopic mechanisms in conventional oxide-based memristors, and the persistent challenges—such as stochastic filament formation and limited multistate controllability—that constrain their broader neuromorphic applicability.

HEOs have emerged as a promising new class of materials capable of addressing these intrinsic limitations. Their entropy-stabilized structures, configurationally distributed defect landscapes, forming-free switching behavior, and intrinsic potential for analog conductance modulation offer unique opportunities for the development of reliable, scalable, and energy-efficient memristive devices. Nonetheless, the complexity of multicomponent chemistries and defect dynamics in HEOs introduces new challenges in controlling switching precision and stability, necessitating further materials engineering and mechanistic understanding.

Building on these insights, the following chapter systematically explores the device architectures, resistive switching characteristics, and neuromorphic functionalities realized in high-entropy oxide-based memristors. By bridging materials design, device operation, and functional performance, these systems are poised to unlock new pathways for brain-inspired hardware technologies.

## Device Architectures and Synaptic Functionality in High-Entropy Oxide Memristors

HEOs with their entropy-stabilized crystal frameworks and tunable defect landscapes offer a unique materials platform for neuromorphic memristive technologies. Moving beyond bulk-level material properties, the practical realization of HEOs in device architectures demands precise control over thin-film synthesis, microstructural engineering, and interfacial modulation to fully exploit their resistive switching capabilities and synaptic-like plasticity.

This chapter focuses on the device-level manifestations of HEO functionalities. We first analyze the structural and electrical advantages conferred by HEO thin films, then discuss the mechanisms underpinning their resistive switching characteristics and multilevel conductance modulation, and finally explore their potential for emulating complex synaptic behaviors in neuromorphic systems.

### Functional Significance of HEO Thin-Film Architectures

#### Dielectric Performance and Structural Stability of HEO Thin Films

While HEOs inherently possess structural stability and tunable electronic properties, their practical deployment in memristive devices hinges on the successful transition from bulk materials to high-quality thin-film configurations. In this regard, HEO thin films serve not merely as carriers of material properties but as critical interfaces that connect entropy-driven material design with device-level performance.

HEO thin films typically exhibit high dielectric constants, low leakage currents, and elevated breakdown field strengths. These attributes arise from the multi-element composition that disperses conduction pathways, suppresses local current filaments, and promotes charge delocalization. The presence of multiple cations facilitates the formation of deep trap states and enhances dielectric stability. For instance, Lan et al. [122] demonstrated that paraelectric HEO films maintaining leakage currents below 10^–6^ A cm^−2^ under electric fields > 8 MV cm^−1^, achieving energy densities exceeding 50 J cm^−3^—favorable for low-power resistive switching and data retention.

Furthermore, the entropy-stabilized chemical homogeneity in HEOs mitigates defect clustering and promotes uniform electric field distribution. Li et al. [123] showed that this structural uniformity suppresses oxygen vacancy aggregation and improves breakdown tolerance, extending device reliability. On the microscopic scale, the complex cation lattice induces symmetry breaking and electronic fluctuations, enhancing dielectric polarizability. Xiong et al. [124] attributed these enhancements to multi-cation-induced electronic delocalization and asymmetric bonding networks, which also support more consistent resistive switching thresholds.

#### Defect Engineering and Interfacial Modulation for Device Reliability

Beyond their intrinsic dielectric advantages, HEO thin films offer a highly tunable defect and microstructural landscape. This tunability has been leveraged to control conductivity, switching uniformity, and long-term stability. For example, Einert et al. [125] synthesized ordered mesoporous (Co, Ni, Cu, Zn, Mg)Fe_2_O_4_ films via sol–gel methods, demonstrating a two-order-of-magnitude conductivity enhancement upon crystallization at 600 °C. Similarly, Tang et al. [126] showed that (La, Pr, Nd, Sm, Eu)NiO_3_ films with a (110) orientation exhibited a sharp metal–insulator transition and a resistance contrast of 2.5 × 10^4^ due to strain-induced band modulation.

Strain-driven effects are further evident in epitaxial (Co, Cr, Fe, Mn, Ni)_3_O_4_ films, where Zhao et al. [127]. observed reversible switching between in-plane and out-of-plane magnetic axes, highlighting the interplay between lattice structure and functional states. Miao et al. [128] advanced this concept by employing temperature-gradient pulsed laser deposition (PLD) to engineer a vertical vacancy gradient in rock salt HEO films, enabling directional conductive filament formation and analog synaptic weight modulation.

Such multilayer defect strategies have also proven effective in non-HEO systems. For instance, Kim et al. [129] employed roughened TiN/HfO_2_ interfaces to localize electric fields and reduce switching variability, achieving forming-free behavior with improved endurance. Similarly, Zhang et al. [130] reported a TiO_2_/HfO_2_ bilayer structure with an hourglass-shaped vacancy profile, compressing variability to ~ 3% and enabling recovery after 10^4^ s at 85 °C under ± 1.6 V cycling. These results provide design templates for extending interface engineering strategies to HEO-based devices.

Recent experiments by Miao et al. [122] further demonstrated that temperature-gradient PLD-fabricated HEOs achieved < 2% write-drift after 10^8^ cycles at 150 °C, confirming their suitability for long-term operation under harsh conditions. These findings collectively validate the role of interfacial and multiscale defect modulation in enhancing HEO device reliability.

Similar structural strategies have also been validated in bio-relevant conditions: for instance, Cao et al. developed oxide-based memristive devices that maintained reliable switching under physiological temperature (37 °C) and mechanical cycling stress > 10 kPa, supporting their potential for wearable and implantable neuromorphic electronics [131].

These multi-scale structural and interfacial strategies not only enhance device-level switching performance and variability control, but also lay the physical foundation for scalable integration, which will be further explored in Sect. 7. In summary, HEO thin films offer a structurally and electronically versatile platform for next-generation memristive systems. Their tunable dielectric properties, programmable defect landscapes, and interfacial adaptability support forming-free switching and efficient synaptic modulation. These traits are expected to bridge nanoscale material design with large-scale integration, paving the way for entropy-engineered neuromorphic hardware.

### Resistive Switching Characteristics of High-Entropy Oxide Memristors

In recent years, memristive systems based on HEOs have demonstrated a range of distinctive resistive switching behaviors, including excellent switching uniformity, enhanced cycling endurance, and finely tunable multilevel conductance states. These properties can be attributed to the synergistic interaction among multiple cations, the tunable distribution of oxygen vacancies, and the modulation of local energy landscapes induced by structural disorder. This section explores the interplay between device architecture, switching mechanisms, and conductance modulation, with the aim of elucidating how HEO materials empower resistive switching dynamics through the coupling of composition, structure, and functionality.

#### Structural and Mechanistic Analysis of Amorphous HEO-Based Memristors

**Fig. 8:**
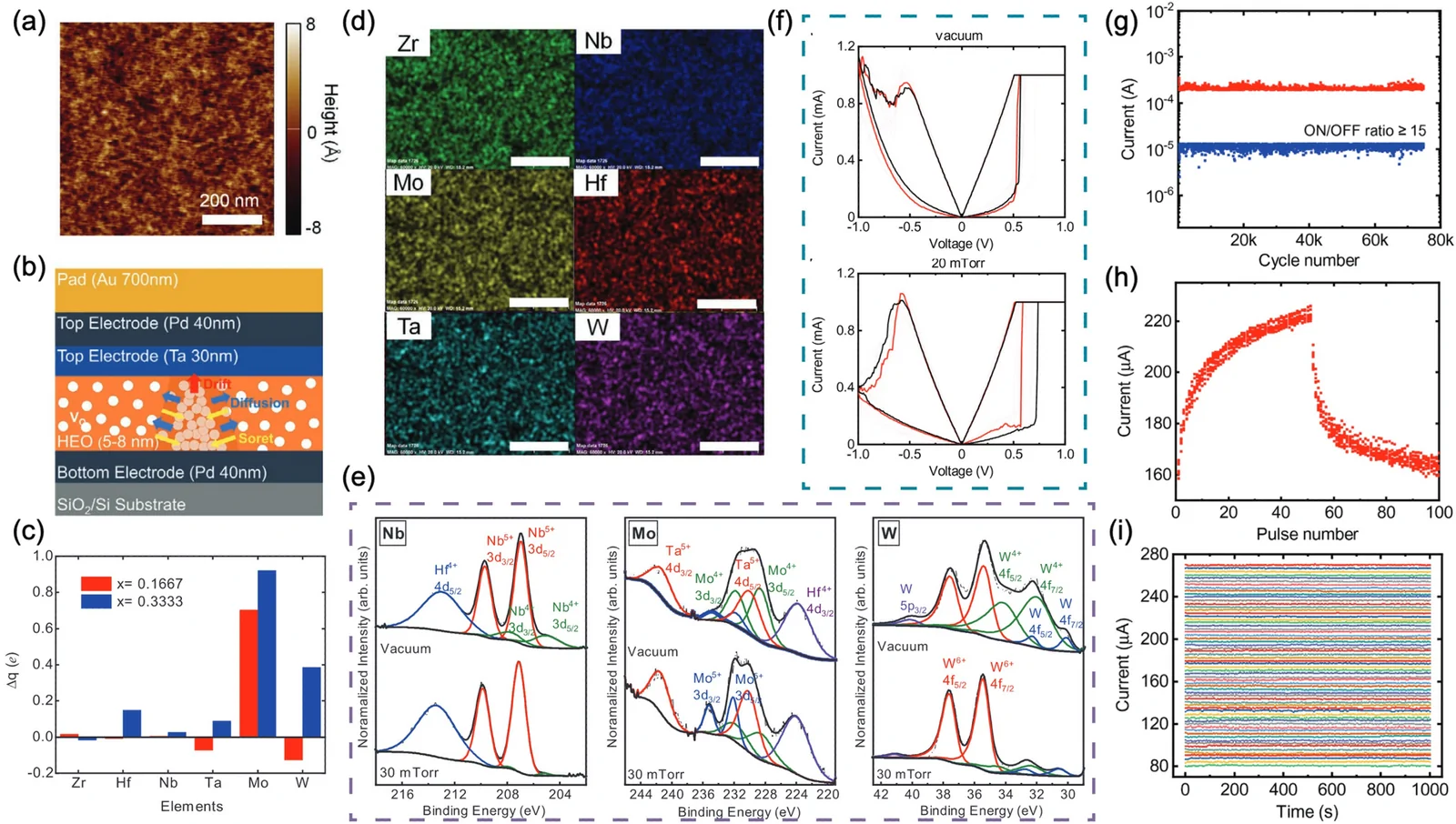
Structural, compositional, and electrical characterization of the amorphous (Zr, Hf, Nb, Ta, Mo, W)_2_O_5−*x*_-based memristor. **a** AFM image showing the atomically smooth surface of the HEO thin film.** b** Schematic illustration of the memristor device structure and conduction mechanism, highlighting drift and Soret diffusion of *V*_O_. **c** Bader charge analysis based on DFT, indicating the dominant charge compensation by Mo and W. **d** EDS elemental mapping of the six cations, confirming homogeneous spatial distribution. **e** XPS spectra under vacuum and 30 mTorr conditions for Nb, Mo, and W, revealing oxygen pressure-dependent valence states. **f** Typical bipolar I–V curves recorded under vacuum and 20 mTorr conditions, demonstrating forming-free resistive switching. **g** Endurance test showing stable ON/OFF switching over 70,000 cycles. **h** Incremental conductance updates under pulse stimulation. **i** Write–verify-controlled 6-bit programming, demonstrating precise and stable multistate retention. Reproduced with permission from Ref. [33], licensed under CC BY 4.0

In 2021, Ahn et al. [33] reported a memristor device based on a six-element HEO system, formulated as (Zr, Hf, Nb, Ta, Mo, W)_2_O_5−*x*_. The device features a Ta/Pd top electrode and a Pd bottom electrode, with the amorphous HEO thin film deposited onto a SiO_2_/Si substrate using PLD (Fig. 8b). Atomic force microscopy (AFM) revealed a remarkably smooth surface with a root-mean-square roughness of approximately 0.109 nm (Fig. 8a), and energy-dispersive spectroscopy (EDS) confirmed a highly uniform elemental distribution across the film (Fig. 8d).

By tuning the oxygen partial pressure during PLD (ranging from vacuum to 30 mTorr), the authors achieved precise control over the concentration of oxygen vacancies. X-ray photoelectron spectroscopy (XPS) results (Fig. 8e) showed that under oxygen-deficient conditions, Mo and W readily formed low-valence suboxides (Mo^4+^, W^4+^), enhancing charge compensation and stabilizing the vacancy network. Bader charge analysis based on first-principles calculations (Fig. 8c) further confirmed the dominant role of Mo and W in the electronic compensation process. This defect-engineering strategy effectively reduced the energy barrier for filament formation and improved switching uniformity.

The device exhibited stable, bipolar resistive switching under both vacuum and low-oxygen conditions: SET transitions (HRS → LRS) occurred under positive bias, and RESET (LRS → HRS) under negative bias, demonstrating reliable reversibility (Fig. 8f). Notably, the device required no electroforming process—resistive switching was initiated in the very first voltage sweep—and consistent performance was observed across multiple devices.

Mechanistically, the switching behavior arises from a synergy between oxygen vacancy migration and local structural rearrangement. Driven by the combined effects of Mott–Gurney drift and Soret thermal diffusion, oxygen vacancies migrate vertically through the film to form localized conductive pathways. The intrinsic “sluggish diffusion” of HEO systems helps homogenize vacancy distribution, mitigating stochastic filament growth and thereby improving switching reproducibility (Fig. 8g, h).

In summary, the device demonstrates multiple advantages—including switching uniformity, multilevel programmability, and low-power operation—highlighting the potential of amorphous HEOs as a material platform for next-generation synaptic electronics.

#### Analysis of Crystalline Spinel-Type HEO-Based Memristors

**Fig. 9:**
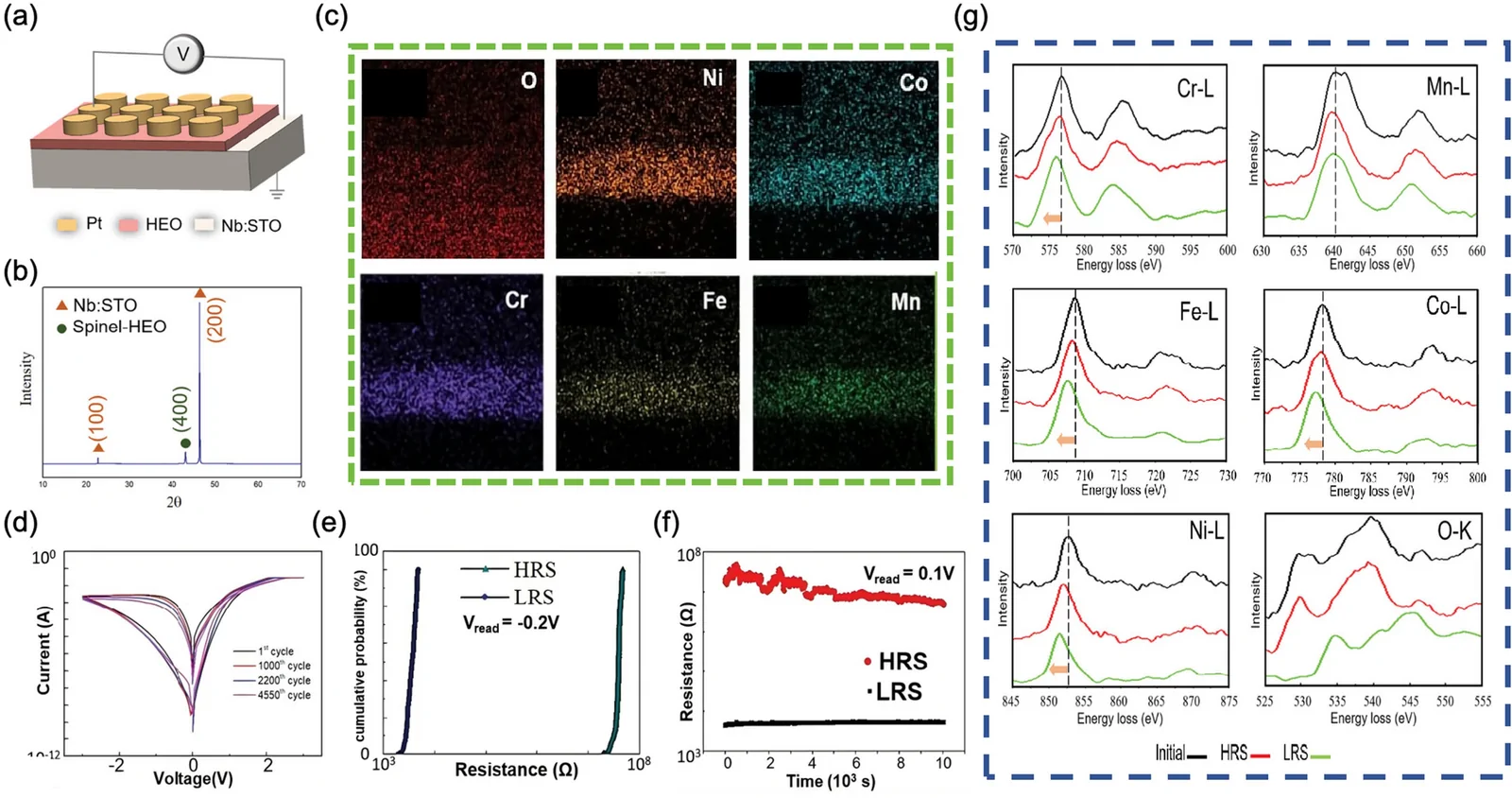
Structural, compositional, and electrical characterization of the crystalline (Cr, Mn, Fe, Co, Ni)_3_O_4_ spinel-type HEO- based memristor. **a** Device schematic.** b** XRD pattern confirming single-phase spinel structure.** c** Elemental mapping of O, Cr, Mn, Fe, Co, and Ni. **d** Bipolar I–V switching behavior over 4550 cycles.** e** Cumulative HRS/LRS resistance distribution. **f** Retention at room temperature under 0.1 V read bias. **g** EELS spectra of Cr–L, Mn–L, Fe–L, Co–L, Ni–L, and O–K edges in pristine, HRS, and LRS states, revealing dynamic valence modulation during switching. Reproduced with permission from Ref. [32], Copyright 2023, John Wiley and Sons

In 2023, Tsai et al. [32] reported a memristor device based on a crystalline HEO with a spinel structure, composed of the multicomponent system (Cr, Mn, Fe, Co, Ni)_3_O_4_. The thin film was epitaxially grown via PLD onto a single-crystal Nb-doped SrTiO_3_ (Nb:STO) substrate, forming a Pt/HEO/Nb:STO sandwich architecture (Fig. 9a). Structural characterization using X-ray diffraction and HAADF-STEM confirmed the high crystallinity and phase purity of the spinel film (Fig. 9b), while EDS mapping verified a homogeneous cation distribution at the atomic scale (Fig. 9c), indicative of a typical high-entropy solid solution.

The device exhibited forming-free, bipolar resistive switching behavior within a stable operating voltage range of ± 3 V, with no electroforming required for reliable SET/RESET cycling.

**Fig. 10:**
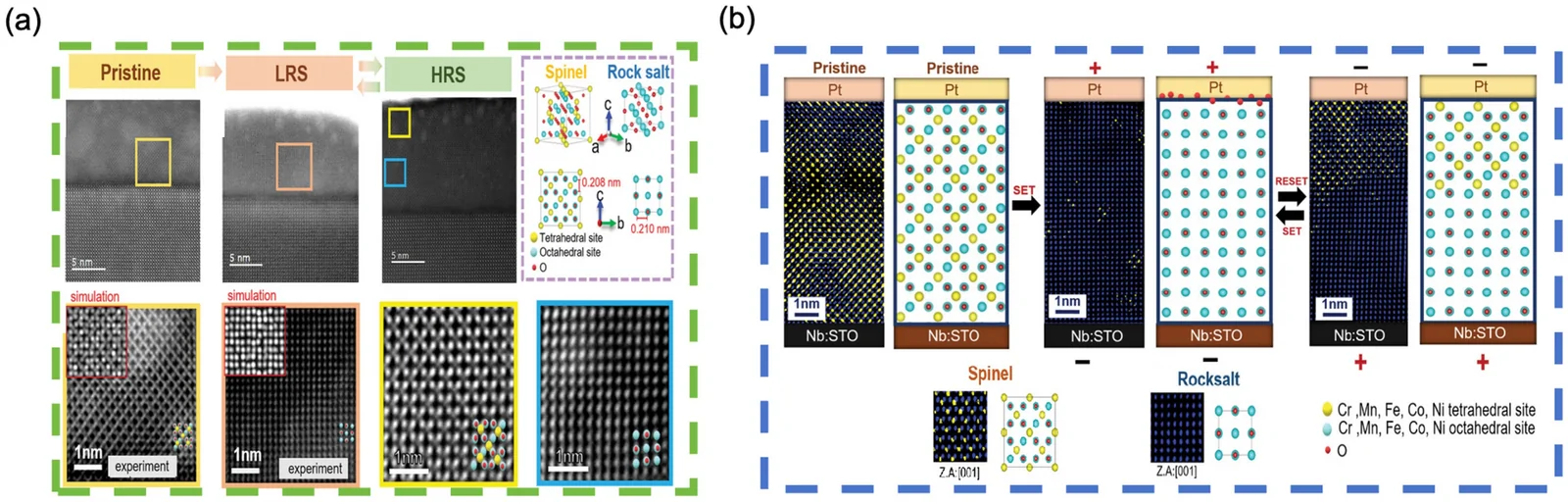
Atomic-scale switching mechanism of spinel-type HEO memristor. **a** STEM images showing phase transition from spinel to rock-salt and partial reversion. **b** Schematic model of the switching pathway involving *V*_O_ migration and structural evolution. Reproduced with permission from Ref. [32], Copyright 2023, John Wiley and Sons

Mechanistically, Tsai et al. proposed a lattice phase transition as the dominant switching pathway: under positive bias (SET), the spinel structure transforms into a more conductive, oxygen-deficient rock-salt phase, accompanied by spontaneous formation of oxygen vacancies (Fig. 10a); under reverse bias (RESET), the structure partially reverts to its original spinel configuration. This reversible transition enables well-defined and spatially confined conductive pathways without reliance on conventional metallic filaments, enhancing both switching stability and controllability.

To further elucidate the evolution of oxidation states during switching, the authors employed EELS and XPS to track the valence changes of multiple transition-metal elements before and after conductive channel formation (Fig. 9g). The analysis revealed that Mn, Fe, and Co undergo a redox shift from + 3 to + 2 during SET, while Cr and Ni partially return to their higher oxidation states during RESET. This behavior reflects a coordinated valence regulation mechanism, in which different cation species contribute either to conductivity enhancement or structural reversibility.

Based on these observations, the authors constructed a mechanistic model integrating three interrelated components: oxygen-vacancy-driven activation, lattice phase transformation, and multi-valence regulation (Fig. 10b). This model captures the essence of the switching process in crystalline HEOs, linking structural evolution with electronic transport pathways.

In summary, the spinel-type HEO device represents a departure from conventional filament-based mechanisms, instead leveraging a phase-transition-dominated switching mode. It exhibits excellent stability, multilevel tunability, and cycling endurance, offering a promising direction for future brain-inspired computing architectures.

#### Mechanistic Abstraction and Mapping to Synaptic Functionality

Although amorphous and crystalline HEO memristors differ markedly in structural order, conduction channel formation, and electronic responses, their resistive switching behaviors are commonly governed by the interplay of three core factors: defect dynamics, lattice structural evolution, and cation valence modulation. Building on the studies by Ahn and Tsai et al., the conduction pathways in both types of systems can be abstracted into generalized mechanistic models and further mapped to functional synaptic behaviors.

In amorphous HEOs, the modulation of conductance primarily arises from the slow migration of oxygen vacancies under the influence of electric fields and thermal gradients, coupled with local structural rearrangement. Owing to the sluggish diffusion characteristic of these materials, the resulting conductive pathways are spatially confined, uniform, and highly reproducible. These features make amorphous devices well suited for implementing gradual, analog-like synaptic weight updates.

In contrast, crystalline HEO devices exhibit resistive switching behavior dominated by field-induced, reversible structural phase transitions. Under positive bias, localized regions of the spinel phase convert into a more conductive rock-salt phase; under negative bias, partial recovery of the original structure occurs, enabling dynamic ON/OFF control. This switching mechanism is triggered by oxygen vacancy formation and accompanied by redox transitions in transition-metal cations, forming a tightly coupled triad of structural transformation, valence evolution, and conductance modulation.

From the perspective of synaptic functionality, amorphous HEO memristors are particularly well suited for mimicking short-term plasticity (STP) and high-resolution weight updates due to their continuous and smooth conductance tuning. Crystalline HEOs, on the other hand, offer clear threshold behavior and memory retention features that align well with long-term potentiation (LTP) and bistable synaptic states.

Collectively, these two classes of devices represent complementary switching modalities: the former supports soft, analog tuning without a defined threshold, while the latter provides hard-switching control with well-defined state transitions. By strategically integrating both types into neuromorphic systems, one can design synaptic architectures tailored to diverse learning rules and computational requirements.

Beyond electrical domain control, recent efforts have explored light-assisted plasticity as an emerging modality to further enrich synaptic tunability. For example, photo-responsive cross-point arrays have demonstrated that optical gating can introduce wavelength-selective potentiation and depression windows [132]. Given the propensity of HEO lattices to host deep-level defect states and exhibit broadband sub-band-gap absorption, similar optoelectronic coupling strategies could be extended to high-entropy oxide systems. This enables multimodal plasticity while preserving the vacancy-dominated switching mechanism—offering new opportunities for adaptive, low-power neuromorphic systems under hybrid electrical–optical control.

### Synaptic Behavior and Multilevel Conductance Modulation in HEO Memristors

Building upon the underlying switching mechanisms, HEO memristors have demonstrated strong potential to emulate key synaptic functionalities, including STP, LTP and fine-grained, programmable conductance modulation. Their tunable resistance states, non-volatility, and structurally responsive behavior render them well suited for neuromorphic computing applications.

In the case of amorphous (Zr, Hf, Nb, Ta, Mo, W)_2_O_5−*x*_ devices reported by Ahn et al., short-duration voltage pulses induced gradual conductance increases followed by slow decay upon stimulus removal—closely mimicking STP-like behavior. Under prolonged stimulation, the devices transitioned into a stable, high-conductance state analogous to LTP. Utilizing a write–verify feedback mechanism, the memristor achieved 64 discrete conductance levels (6-bit resolution), with per-step current deviation limited to ± 0.25 μA (Fig. 8i), enabling precise and continuous synaptic weight updates.

Crystalline devices based on (Cr, Mn, Fe, Co, Ni)_3_O_4_, by contrast, leverage a phase-transition-driven switching mechanism to realize different plasticity behaviors. By modulating pulse amplitude, duration, and compliance current, these devices support continuous conductance tuning, enabling the emulation of spike-timing-dependent plasticity (STDP) and other temporally coded learning rules. The intrinsic reversibility of the structural transition further supports stable state retention and consistent response characteristics, positioning these devices as viable candidates for long-term memory storage.

In terms of electrical stability, both device types exhibit excellent retention and endurance. The amorphous HEO devices maintained their conductance state for over 20 h at 100 °C, with endurance exceeding 7 × 10^4^ cycles. Crystalline counterparts retained stable states for over 10^4^ s at room temperature and supported more than 4500 reliable switching cycles (Fig. 9d–f). These robust characteristics are attributed to the defect coordination and multivalent ion regulation inherent in HEO materials.

Targeted thermal stress and cycling measurements provide a clearer picture of device reliability. Amorphous (Zr, Hf, Nb, Ta, Mo, W)_2_O_5−*x*_ devices keep their resistance levels unchanged for more than 20 h at 100 °C; Arrhenius scaling indicates data retention approaching ten years at 85 °C. They also tolerate 7 × 10^4^ switching cycles under ± 1 V, with device-to-device spread of roughly 8%. Spinel (Cr, Mn, Fe, Co, Ni)_3_O_4_ devices, operated forming-free within ± 3 V, show almost no change in I–V curves when cycled between –10 and 90 °C, and their conductance drifts by less than 10% after 60 days in air while surviving about 4.5 × 10^3^ cycles. These results suggest that a dense, entropy-stabilized vacancy network in the amorphous film, together with reversible multivalent-ion phase transitions in the crystalline film, underpins the observed thermal stability and analog uniformity. Even so, pushing σ/μ below 10% for more than 10^5^ cycles under elevated-temperature stress will require additional compositional and layout refinement. +

Power efficiency and array-scale potential are equally encouraging. The amorphous high-entropy-oxide device based on (Zr, Hf, Nb, Ta, Mo, W)_2_O_5−*x*_ switches reproducibly at about ± 1 V, whereas the spinel counterpart, (Cr, Mn, Fe, Co, Ni)_3_O_4_, requires only ± 3 V; neither architecture demands an electroforming step. Such low-field, structure-guided conduction points to sub-picojoule switching energy and favors dense cross-bar integration, although direct fJ-per-bit measurements have yet to be reported.

Even at this stage, the two devices already deliver the key synaptic functions: short- and long-term plasticity, 64-level analog weight tuning in the amorphous device, and two-month ambient stability with < 10% drift, together with resilience to − 10 to 90 °C temperature sweeps and extensive write–erase cycling. Moving forward, advances in composition control, stack engineering and algorithm–hardware co-design should push endurance beyond 10^5^ cycles, tighten *σ*/*μ* below the 10% benchmark, and drive the switching energy firmly into the femtojoule regime—milestones that would place HEO memristors on a competitive footing for large-scale neuromorphic arrays.

## Toward Multi-Scale Integration of High-Entropy Oxide Memristors in Brain-Inspired Systems

HEO memristors have demonstrated promising device-level capabilities—including forming-free switching, multilevel conductance tuning, and synaptic plasticity emulation. However, leveraging these features at the system level remains a nontrivial challenge. While previous chapters have addressed the materials basis and switching mechanisms in detail, the critical next step lies in understanding how these physical properties can be faithfully translated into reliable, scalable, and programmable behavior within large-scale neuromorphic systems.

### Coupling Tension Between Material Response and System Requirements

Although HEO memristors offer considerable physical flexibility, including rich defect chemistry and structural reconfigurability, these same features can also introduce unpredictable variations—especially when deployed in dense crossbar arrays. Unlike isolated device testing, neuromorphic networks require tight control over conductance precision, weight stability, and endurance across thousands or millions of devices operating in parallel.

To date, most studies have focused on validating synaptic behaviors at the individual device level, such as STP, LTP, and multistate modulation. However, network-scale applications demand additional metrics: symmetric and linear weight updates, robust state retention under stress, and minimal drift during prolonged use [133]. Many of these factors remain unaddressed in current HEO-based systems. Additionally, the intrinsic stochasticity from cationic disorder and local strain fields can lead to significant device-to-device variability, manifesting as accumulated noise in large arrays and impeding convergence in training tasks.

Addressing this cross-scale inconsistency requires a systemic shift—from demonstrating individual behaviors to ensuring holistic performance alignment. Material complexity must be reconciled with system predictability, and entropy-enhanced functionality must be embedded within well-characterized operational envelopes. Moreover, HEO devices must be evaluated not just for their intrinsic capabilities, but for how reliably those capabilities scale with task complexity and system constraints.

Recent demonstrations on 128 × 128 selector-free HEO crossbar arrays (*σ*/*μ* ≈ 8%) [33] serve as a practical benchmark. To this end, we propose a mid-term integration target of *σ*/*μ* ≤ 5%, achievable through co-integration of vacancy-buffering interlayers, thermally graded vacancy stacking, and architectural redundancy. For example, the insertion of ~ 2 nm TiO_2_ or Al_2_O_3_ buffer layers has previously reduced HfO_2_-based RRAM variability to ≈ 3% [130]; thermal-gradient PLD stacking further suppresses write drift below 2% over 10^5^ cycles at 150 °C [128]. Combined with column-level fault tolerance and selector-compatible fabrication (≤ 300 °C), these methods outline a feasible pathway to < 5% variability in full-array contexts.

The following sections shift the focus toward system-level mapping strategies and architectural compatibility, outlining how material-level advances in HEO memristors can be translated into functional building blocks for neuromorphic computing.

### Building a Multi-Scale Mapping Framework

This section lays out a hierarchical mapping framework that connects the unique physical behaviors of HEO memristors with the performance requirements of practical computing systems, spanning device, architecture, and task levels.

To transition HEO memristors from material innovations to computational workhorses, a coherent multiscale mapping framework is essential. This framework must bridge three hierarchical levels:

- **Device–Precision Alignment**: While HEO devices offer analog tunability, maintaining low variability and ensuring symmetric write–erase dynamics are critical for gradient-based learning. Research by Aguirre et al. [134] shows that asymmetric or nonlinear conductance updates can degrade training accuracy and hinder convergence. Therefore, future efforts must not only increase the number of conductance states but also ensure their fidelity, reproducibility, and linear accessibility.
- **Architecture–Robustness Co-Design**: Local inhomogeneities in HEO thin films—such as vacancy clustering or strain gradients—can translate into global instability if unmitigated. Redundancy-aware design, localized recalibration, and dropout-inspired architectures can act as statistical "shock absorbers" that decouple hardware noise from network-level error propagation [135–137]. These strategies, drawn from fault-tolerant computing, offer a practical path toward robust large-scale deployment. A recent demonstration of a laser-programmed phase-change photonic CNN shows that hardware-level “dropout”––achieved by randomly freezing 10–15% of optical synapses––can suppress gradient noise and recover full software accuracy even when unit-to-unit variation exceeds 7% [138]. The same redundancy-aware principle can be migrated to HEO crossbars by combining column-level sparing with on-chip re-training, effectively absorbing the residual 5% device spread projected for *σ*/*μ*-optimized HEO arrays.
- **Mechanism–Task Compatibility**: Different switching mechanisms intrinsic to HEOs—oxygen vacancy migration, phase transitions, or valence modulations—may align better with some neural network architectures than others. For instance, spiking neural networks require precise temporal encoding and rapid plasticity, whereas transformer-based architectures prioritize analog resolution and long-term retention. Tailoring pulse schemes, write protocols, and device operating conditions to specific learning rules is critical for system-level compatibility.

Together, these layers form a functional roadmap for guiding the integration of HEO memristors into neuromorphic hardware. Rather than focusing solely on material capability, the field must increasingly address how these capabilities scale, interact, and contribute to usable, trainable systems.

### HEO Memristors toward Emerging Neuromorphic Architectures: Integration Challenges and System-Level Opportunities

The rapid advancement of neuromorphic hardware—from selector-assisted 1S1R arrays and 3D TSV stacking, to integrated on-chip spiking neural networks (SNNs), transformer-in-memory architectures, and near-sensor computing platforms—has introduced stringent performance and integration criteria for memristive components [139, 140]. Essential benchmarks include (i) CMOS BEOL compatibility below 300 °C, (ii) forming-free operation within ± 0.5–1 V, (iii) excellent thermal and cycling stability, and (iv) minimized variability across large-scale arrays [139–142].

While HEO memristors are predominantly at the single-device validation stage, several pioneering studies already demonstrate promising compliance with these requirements. Amorphous (Zr, Hf, Nb, Ta, Mo, W)_2_O_5−*x*_ devices fabricated at room temperature achieve forming-free resistive switching at ± 1 V, with device-to-device variability currently around 8% [33]. Spinel-type (Cr, Mn, Fe, Co, Ni)_3_O_4_ memristors exhibit forming-free switching within ± 3 V and retain an ON/OFF ratio greater than 10^3^ for over 10^4^ s even at elevated temperatures of 175 °C, highlighting their inherent robustness against thermal stress [32].

However, significant integration challenges remain for HEO-based devices, particularly regarding variability and large-scale uniformity. Although HEOs inherently provide distributed and entropy-stabilized defect landscapes, the complexity arising from multi-cation distributions introduces considerable uncertainty in defect migration pathways and activation energies. This complexity complicates the deterministic control of switching thresholds and the precise modulation of conductance levels critical for large-scale arrays. Additionally, the coupling between defect dynamics, structural phase transitions, and local redox processes further complicates accurate predictive modeling and engineering of stable neuromorphic performance.

Beyond purely electrical performance metrics, the unique optoelectronic and multifunctional properties of HEOs may significantly enhance heterogeneous integration opportunities. Recent developments in CMOS-compatible AlN/Si photonic cores, achieving sub-10 ns latency at only 0.34 pJ/MAC [143], align seamlessly with the low-voltage and potentially ultrafast switching characteristics of HEO memristors. Moreover, envisioning multimodal sensor integration (pressure, optical, thermal) at the memory-sensor interface could directly encode environmental stimuli into synaptic pulses, thereby eliminating costly analog-to-digital conversion steps and further reducing system latency and power consumption [144].

Despite promising single-device performance, the transition of HEO memristors to fully integrated neuromorphic platform necessitates rigorous validation at the array level, focusing on energy-efficient operation, long-term cycling endurance, and inherent hardware security. Here, we highlight several prototypical integration paradigms to facilitate future developments:

- **Pixel-Level Vision-in-Memory Architectures:** Utilizing a high-entropy alloy (Cu, Al, Ag, Cr) top electrode, the TiO_2_/W-based optoelectronic memristor demonstrates visible-light-induced photo-potentiation spanning nanoampere to microampere currents, reversible volatile and non-volatile resistive switching, and in situ Boolean logic operations such as AND. These features enable a compact “pixel-level sensing-storage-computing” modality conducive to event-driven neuromorphic vision processing [145].
- **Secure Edge Inference Modules:** Polymer-based pV3D3 memristor arrays realize physically unclonable functions (PUFs), yielding cryptographic keys resilient against adversarial machine learning attacks [146]. Substituting the polymer matrix with entropy-stabilized oxides promises to retain intrinsic entropy sources while significantly improving thermal stability, thereby embedding robust hardware-rooted security primitives within HEO memristor crossbar arrays.
- **Printable, Flexible Neuromorphic Substrates:** Inkjet- and microplot-printed high-entropy Prussian blue analog (HE-PBA) memristors operate forming-free at sub-0.3 V voltages, deliver a high resistance window (*R*_OFF_/*R*_ON_ ≈ 10^4^), and maintain microwatt-scale power consumption, collectively underpinning cost-effective, large-area flexible neuromorphic skins suitable for scalable sensory interfaces [147].

In summary, the intrinsic material advantages of HEO memristors—such as their low-temperature synthesis, forming-free switching capability, and tunable multilevel conductance—position them as highly promising building blocks for next-generation neuromorphic architectures. To realize their full potential, continued progress in material optimization, robust selector-device co-design, precise thermal management, and advanced array-level fault tolerance strategies is essential. With concerted efforts across these fronts, large-scale deployment of HEO-based neuromorphic systems is becoming increasingly attainable, promising a significant leap forward for brain-inspired computing technologies.

## Conclusion and Outlook

HEO memristors have emerged as a compelling materials platform for neuromorphic electronics, distinguished by their inherent configurational entropy, tunable defect chemistry, and structural complexity. This review has constructed a comprehensive multiscale framework encompassing entropy-driven phase stabilization, defect and interface engineering, resistive switching mechanisms, and system-level integration, thereby elucidating the intrinsic advantages of HEOs over conventional memristive materials.

Through the incorporation of diverse multivalent cations and oxygen nonstoichiometry, HEOs facilitate forming-free resistive switching and robust multilevel conductance modulation within both crystalline and amorphous frameworks. Such versatility enables the emulation of diverse synaptic behaviors—ranging from analog gradual potentiation to discrete abrupt switching—positioning HEO devices as promising candidates for adaptive and energy-efficient neuromorphic hardware. Furthermore, the compatibility of HEO thin films with low-temperature, scalable fabrication techniques renders them highly suitable for BEOL integration, flexible electronics, and hybrid photonic–electronic systems.

Despite significant advances at the device level, critical obstacles persist in scaling HEO memristors for system-level deployment. Principal challenges include mitigating conductance drift, suppressing device-to-device variability, and enhancing linearity and symmetry in synaptic weight updates. These limitations constrain the direct translation of material-level improvements into algorithmic robustness and hardware performance.

Overcoming these challenges demands progress across several interrelated domains:

- **Entropy-Guided Material Design:** Transitioning from empirical compositional exploration toward a rational defect–structure–property paradigm is essential. Optimizing configurational entropy, cation valence distributions, and lattice disorder will underpin reproducible and stable switching characteristics.
- **Variability Suppression Via Interface and Structural Engineering:** As detailed in Sects. 6.3 and 7.3, techniques such as gradient-controlled deposition (e.g., temperature-programmed pulsed laser deposition), implementation of ultrathin vacancy-buffering interlayers (e.g., TiO_2_, Al_2_O_3_), and electric field homogenization are critical to constraining variability metrics (σ/μ) below 5%, while preserving endurance and thermal robustness.
- **Scalable, CMOS-Compatible Processing and Hybrid Integration:** Demonstrations of low-voltage (< 0.3 V), forming-free operation and ultra-low energy consumption (fJ–pJ per bit) in conjunction with multimodal sensing and photonic neuromorphic modules underscore the potential of HEOs for large-area, flexible, and heterogeneous neuromorphic platforms.
- **Device-Algorithm Co-optimization and Architecture-Aware Design:** As explored in Sect. 7.2, integrating materials insights with neural network training algorithms—through redundancy-aware learning, fault-tolerant architectures, and adaptive calibration—facilitates the mitigation of device stochasticity, thereby enhancing large-scale system reliability.

Recent efforts have also marked a shift from empirical compositional screening to structure–defect–property-guided design strategies. This evolution has coincided with the growing potential of HEO-based memristors beyond classical neuromorphic computing, including emerging domains such as edge AI, in-sensor processing, and multimodal perception platforms. Specifically, entropy-enabled multifunctionality—spanning thermoelectric, piezoelectric, and optoelectronic coupling—positions HEOs as a versatile class of materials for next-generation brain-inspired hardware.

Ultimately, the true impact of HEO memristors may not lie in incremental improvements to existing device metrics, but in their ability to redefine how complex materials can support intelligent computation. Rather than treating structural disorder and configurational complexity as limitations, future research may embrace them as new engineering degrees of freedom for adaptability, plasticity, and energy efficiency. In this light, HEO memristors are not merely high-performance memory elements—they represent a material-centric framework for rethinking the architecture of brain-like electronic systems.
